# Genome-wide identification and characterization of CKIN/SnRK gene family in *Chlamydomonas reinhardtii*

**DOI:** 10.1038/s41598-018-35625-8

**Published:** 2019-01-23

**Authors:** Francisco Colina, Joana Amaral, María Carbó, Gloria Pinto, Amadeu Soares, María Jesús Cañal, Luis Valledor

**Affiliations:** 1 Plant Physiology, Department of Organisms and Systems Biology and University Institute of Biotechnology (IUBA), University of Oviedo, Oviedo, Spain; 2 Department of Biology and CESAM, University of Aveiro, Aveiro, Portugal

## Abstract

The SnRK (Snf1-Related protein Kinase) gene family plays an important role in energy sensing and stress-adaptive responses in plant systems. In this study, Chlamydomonas CKIN family (SnRK in Arabidopsis) was defined after a genome-wide analysis of all sequenced Chlorophytes. Twenty-two sequences were defined as plant SnRK orthologs in Chlamydomonas and classified into two subfamilies: CKIN1 and CKIN2. While CKIN1 subfamily is reduced to one conserved member and a close protein (CKIN1L), a large CKIN2 subfamily clusters both plant-like and algae specific CKIN2s. The responsiveness of these genes to abiotic stress situations was tested by RT-qPCR. Results showed that almost all elements were sensitive to osmotic stress while showing different degrees of sensibility to other abiotic stresses, as occurs in land plants, revealing their specialization and the family pleiotropy for some elements. The regulatory pathway of this family may differ from land plants since these sequences shows unique regulatory features and some of them are sensitive to ABA, despite conserved ABA receptors (PYR/PYL/RCAR) and regulatory domains are not present in this species. Core Chlorophytes and land plant showed divergent stress signalling, but SnRKs/CKINs share the same role in cell survival and stress response and adaption including the accumulation of specific biomolecules. This fact places the CKIN family as well-suited target for bioengineering-based studies in microalgae (accumulation of sugars, lipids, secondary metabolites), while promising new findings in stress biology and specially in the evolution of ABA-signalling mechanisms.

## Introduction

Algae are promising organisms for biotechnological applications, ranging from the production of biofuels or feedstock to the accumulation of high value-added molecules. This, together with its ease of cultivation, simple life cycle, high growth rate, phenotypic plasticity, and capability to accumulate biofuel-related molecules (lipids, starch) or secondary metabolites such as ß-carotene, astaxanthin or Omega-3^1^ make this group of organisms one of the most studied biological systems.

However, several studies report that the accumulation of these molecules requires the imposition of an external stress. For instance the accumulation of TAG (triacylglycerides), essential for biodiesel production, is mainly triggered by nutrient limitation^2–5^. The accumulation of other valuable molecules such as carotenes^6^ or hydrogen^7^ is also dependent on nutrient or environmental stresses. This need for stressing algae cultures forces a two-phase cultivation strategy in which cells are first grown under optimal conditions until enough biomass is produced and then an abiotic stress is imposed to trigger the accumulation of specific molecules^8,9^. This approach requires longer cultivation times and/or the expense of energy to apply a certain stress (e.g. removing a nutrient from the media), making this production method economically unsustainable^10^.

Studying metabolic and regulatory networks involved in stress response is thus a mandatory first step to identify targets with potential to improve microalgae strains and culture practices^11^. Among the possible cellular mechanisms proposed as targets for microalgae production enhancement, there is evidence that the cAMP-dependant SnRK1 protein kinase family [Sucrose non-fermenting-1 (Snf1)-related protein kinase; known as Snf1 in yeast/mammals and AKIN in *Arabidopsis thaliana*] mediates the connexion between central metabolism, gene regulation, and stress response, together with hexokinases (HXK) and Sucrose-Phosphate Phosphatase (SPP)^12,13^. This protein family is also interconnected with TOR^14^, epigenetic pathways^15^ and with the activation/repression of entire metabolic branches in other organisms^16^, making it a good candidate for further studies in microalgae.

The first described SnRK family member was the yeast Snf1, being well-known by its role in lipid accumulation^17^ and glucose repression, regulating carbon metabolism^12^. Globally, AKIN/SnRK1/Snf1/AMPK kinases concentrate divergent stress signals by activating specific enzymes and transcription factors, related to metabolic regulation, protein biosynthesis and cell organization, as part of a complex system aimed to increase cell survival under unfavourable energetic balance^13,18^. AMPK regulates through direct phosphorylation or associated γ subunits ADP-AMP levels sensing^19^, with also associated β subunits regulating substrate specificity and cell location^20^. These kinase-regulatory subunits protein complexes are remarkably well conserved across Eukarya and deeply rooted into the life tree. Orthologs to AMPK and γ subunits have been found into Bacteria and Archaea kingdoms, being β subunits exclusive from eukaryotes^21^. Plants are an exception of this family conservation showing unique γ functionally equivalent βγ proteins and γ-like subunits without direct SnRK1 interaction^22^. This exceptionality comes also at regulation level, with a sugar phosphate mediated regulation of the energy sensing kinase^23^. Moreover, although AMPK duplication events are common in plants and animals^21^, plants SnRK family underwent an extensive duplication and diversification event, giving origin to three subfamilies: SnRK1/AKIN (the closest to Snf1), SnRK2 and SnRK3^24^. All these plant SnRK subfamilies share a common Ser/Thr kinase domain, followed by a UBA and a KA1 domain in SnRK1. In SnRK2 an osmotic stress activation domain I is present after the kinase domain, while SnRK3 contains a NAF/FISL domain^25^. There is strong evidence that subfamilies SnRK2 and 3 evolved after gene duplication of SnRK1 in order to enable plants to develop networks capable of linking stress, ABA, and calcium signalling with metabolic and epigenetic responses^13^. In plants, some SnRK2 are also key transductors in ABA-mediated responses to salt and other abiotic stresses^25^, presenting specific ABA interaction acidic motifs known as domain II^26^ placed after the characteristic osmotic stress activation domain I.

In *Arabidopsis thaliana*, thereafter Arabidopsis, the SnRK family is composed by 38 members and 3 subfamilies: SnRK1 (3 genes), SnRK2 (10 genes), and SnRK3 (25 genes)^27^. SnRK1 has been linked to increased tolerance to nitrogen stress^28^ and to energy sensing and gene regulation^14^. It showed to be implied in plant response to starvation and energy deficit by coordinating ATP/cAMP, redox status and C/N ratios to regulate broad metabolic branches, either directly by phosphorylation of enzymes or indirectly by phosphorylation of transcription factors^13,29,30^. SnRK1 has been considered a potential target to improve plant performance under unfavourable conditions^25^. SnRK2 and SnRK3 showed to have also a key role in signalling pathways that regulate plant response to nutrient limitation, drought, cold, salt, and osmotic stress^25^. The SnRK2 subfamily has an essential role in gene expression regulation through the activation of bZIP transcription factors and SWI/SNF/helicase complexes^12,31^ tightly connected to epigenetic mechanisms that perfectly control gene activation or repression. However, little is known about the structure or the role of this protein kinase family in microalgae stress response mechanisms and its relation with biotechnological processes, such as the accumulation of high value-added molecules like energetic molecules (sugars and lipids)^32^ or pigments (astaxanthin, lutein, and β-carotene).

The Chlorophyceae *Chlamydomonas reinhardtii*, thereafter Chlamydomonas, shares common ancestry with vascular plants^33^. Therefore, it is expected that many of its responses to limiting conditions would be similar^34^. Few studies suggest the involvement of SnRK family, named CKIN in Chlamydomonas^35^, in stress response, namely under sulphur^36,37^ and nitrogen deprivation^5^, and cold stress^35^. Gonzalez-Ballester *et al*.^36^ reported the existence of eight putative SnRK2-like genes in Chlamydomonas, while Valledor *et al*.^35^ found three cold stress-responsive proteins showing sequence homology with Arabidopsis AKIN10/11 family (CKIN1, CKIN2, and CKIN3). Both authors suggested that, like in plants, Chlamydomonas abiotic stress response is mediated by CKINs. Notably, it has been recently reported by Sato *et al*.^32^ that *SAC1* and *SnRK2*.*2* act as positive regulators of DGTT1, enhancing TAG synthesis under Sulphur starvation in Chlamydomonas. Chlamydomonas SnRKs similarity to land plants is conceivably not only limited to direct stress SnRK induction, but also ABA-SnRK interaction. ABA showed to be involved in Chlamydomonas cell signalling during osmotic stress response^38^. However, microalgae ABA-mediated responses seem to be less complex than in land plants as little or no homology was found between most land plants ABA receptors/effectors and the Chlamydomonas proteome^39,40^. Considering that SnRKs control entire branches of the metabolism in Arabidopsis and other studied models, the identification of CKIN stress-specific dynamics, will potentially reveal new targets for further bioengineering research aiming to accumulate economically relevant biomolecules.

Therefore, in the present study, we aim to fully describe the entire set of genes belonging to the CKIN family in Chlamydomonas and its potential implication in specific stress response mechanisms and in ABA-mediated responses. The combination of Chlamydomonas and other microalgae genome mining, plant protein-protein interaction databases, and quantitative reverse transcription PCR (RT-qPCR) allowed not only the definition of this family and its evolutive history, but also defining its interacting networks and testing its expression levels under exogenous ABA addition, ABA synthesis inhibition, and a wide-range of stressful conditions. The results herein presented represent a great advance in microalgae and stress biology research, defining a new set of potential targets for biotechnological improvement. Although SnRK are a key group of protein kinases for biotechnology, this family was never fully characterized in microalgae.

## Results

### Identification of SnRK protein orthologs in Chlamydomonas

Initial BLAST searches against Chlamydomonas genome employing Arabidopsis SnRKs and identified Chlamydomonas CKIN sequences (Supplementary Table S1) as queries determined 110 proteins that showed significant homology to this family (e-value < 10^−25^; Supplementary Table S2). Calcium Dependent Protein Kinases (CDPKs), CKINs, and other proteins were present in this initial set due to the conserved Ser/Thr kinase domain. Further analyses of protein domains allowed the unequivocal distinction between CKINs and other proteins attending to other domains specifically present in this family (UBA, KA1/αCTD, CBS, Immunoglobulin E-set/CBM and βCTD/ASC/AMPKβI). The combination of BLAST and protein domain validation resulted in the identification of 19 putative CKIN sequences. Furthermore, manual analysis of genome employing domain family annotations present in BIOMART database allowed the determination of three new sequences, making a total of 22 sequences (Table 1). Out of these, 10 genes were previously described by Gonzalez-Ballester *et al*.^36^ and Valledor *et al*.^35^, while 12 were found in Chlamydomonas for the first time. Protein alignment, phosphorylation site identification, functional prediction of unannotated domains, expression and protein interaction analyses were performed for the curation of the identified sequences as described below.

**Table 1:** *Chlamydomonas reinhardtii* (*Cre*) BLAST (e-value < 10^−25^) and BIOMART SnRK/CKIN sequence search hits.

*Cre* Accession	*Ath* Accession	e-value	Name	Cluster	Domain ID
Cre04.g211600.t1.1	AT3G01090.1	0	CKIN1*^a^	CKIN1	PTHR24343:SF183 IPR015940 IPR001772
Cre13.g570250.t1.1	AT1G78290.2	5.96e^−48^	CKIN1L	CKIN1	PTHR24346:SF5 IPR015940
Cre10.g457500.t1.1	AT4G16360.1	1.86e^−69^	CKIN β	S1 R (β)	PTHR10343 IPR032640 IPR006828
Cre12.g484350.t1.3	AT1G09020.1	2.88e^−52^	CKIN βγ	S1 R (βγ)	PTHR13780:SF35 IPR032640 IPR000644
Cre12.g528000.t1.2		Domain	CKIN γ*^a^	S1 R (γ)	PTHR13780:SF49 IPR000644 IPR013785
Cre02.g075850.t1.1	AT4G33950.1	1.33e^−57^	CKIN2.1*^b^	CKIN2	PTHR24343
Cre12.g499500.t1.1	AT1G78290.2	4.02e^−117^	CKIN2.2*^b^	CKIN2	PTHR24343:SF207
Cre02.g075900.t1.1	AT5G66880.1	6.60e^−73^	CKIN2.3*^b^	CKIN2	PTHR24343
Cre11.g477000.t1.2	AT5G08590.1	7.14e^−29^	CKIN2.4*^b^	CKIN2	PTHR24343
Cre03.g209505.t1.1	AT1G78290.2	2.60e^−63^	CKIN2.5*^b^	CKIN2	PTHR24343
Cre11.g481000.t1.2	AT4G33950.1	1.91e^−88^	CKIN2.6*^b^	CKIN2	PTHR24343
Cre06.g292700.t1.2	AT4G33950.1	8.68e^−103^	CKIN2.7*^b^	CKIN2	PTHR24343
Cre10.g466350.t1.1	AT4G33950.1	2.96e^−152^	CKIN2.8*^b^	CKIN2	PTHR24343:SF169
Cre16.g657350.t1.2	AT1G78290.2	5.77e^−59^	CKIN2.9	CKIN2	PTHR24343
Cre17.g707800.t1.2	AT1G78290.2	9.81e^−46^	CKIN2.10	CKIN2	PTHR24343:SF167
Cre02.g076000.t1.2	AT5G63650.1	2.10e^−40^	CKIN2.11	CKIN2	PTHR24343
Cre17.g707650.t1.1		Domain	CKIN2.12	CKIN2	PTHR24343:SF167
Cre12.g485600.t1.2	AT1G78290.2	5.64e^−47^	CKIN2.13	CKIN2	PTHR24343:SF167
Cre13.g568050.t1.3	AT4G33950.1	4.77e^−58^	CKIN2.14	CKIN2	PTHR24343
Cre08.g384250.t1.2		Domain	CKIN2.15	CKIN2	PTHR24343
Cre07.g329850.t1.1	AT5G63650.1	4.69e^−37^	CKIN2.16	CKIN2	PTHR24343
Cre16.g685389.t1.1	AT1G78290.2	6.54e^−33^	CKINL^*a^	CKIN2	PTHR24343:SF200

*Cre* and *Arabidopsis thaliana* (*Ath*) orthologs sequence accessions, homology search e-values (not for exclusive BIOMART hits), *Cre* sequence names, and domain identifiers (ID) provided. CKIN were grouped according to sequence similarity and protein domains layout into different clusters (CKIN1: containing the Serin/Threonin Kinase PTHR24343:SF183, IPR015940 and IPR001772 domains; S1 R: regulatory subunits of CKIN1 with PTHR10343, PTHR13780:SF35, IPR032640, IPR000644, IPR006828 and IPR013785 domains; CKIN2: containing the Serin/Threonin Kinase PTHR24343, PTHR24343:SF169, PTHR24343:SF207, PTHR24343:SF167 or PTHR24343:SF200 domains, with CKIN1L exception, containing the MAP/microtubule affinity-regulating kinase PTHR24346:SF5 and IPR015940 domains). *Chlamydomonas sequences previously referred to as CKIN family by ^a^Valledor *et al*. (2013) and CKIN2 subfamily ^b^by Gonzalez-Ballester *et al*. (2008).

**Figure 1:**
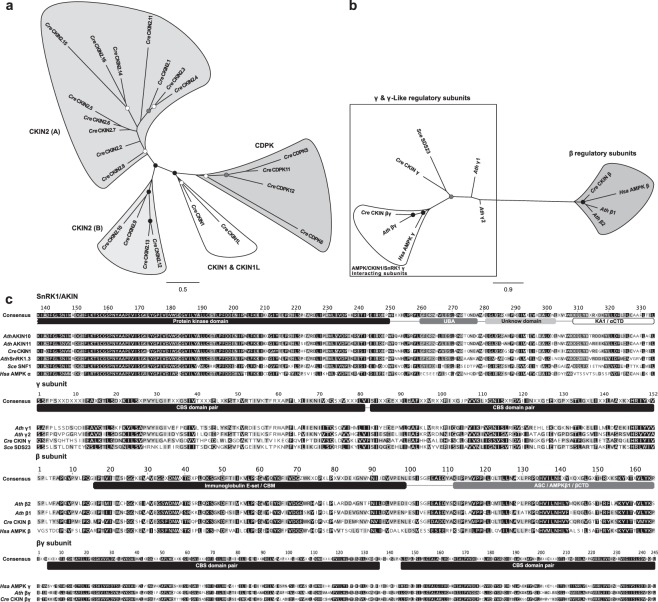
M-Coffee based sequence clustering and structural analysis of *Chlamydomonas reinhardtii* (*Cre*) CKIN sequences. Tree (**a**) arranged catalytic CKIN sequences into 3 clusters, namely CKIN2 (A), CKIN2 (B) and CKIN1 & CKIN1L, including a fourth CDPK cluster. Tree (**b**) included all *Cre* SnRK1/CKIN1 regulatory interacting and related sequences along with *Arabidopsis thaliana* (*Ath*), *Homo sapiens* (*Hsa*) and *Saccharomyces cerevisiae* (*Sce*) orthologues. Regulatory sequences group into two clusters, namely β regulatory subunits and γ and γ-Like regulatory subunits. This second cluster differentiates between CKIN1/SnRK1/AKIN/AMPK interacting subunits and γ-Like non-interacting subunits. FBP bootstrap support for both trees are indicated through color circles over nodes (black: >90, grey: >80, white: >70). (**c**) M-Coffee curated alignments of CKIN1/SnRK1/AKIN and associated regulatory protein clusters. *Cre* sequences show same domain structure as seen on *Ath*, *Sce* and *Hsa* SnRK1/SNF1/αAMPK being *Hsa*AMPK the more dissimilar one.

M-Coffee alignment of 4 Chlamydomonas CDPK along the 19 identified Chlamydomonas catalytic CKIN sequences allowed the definition of three putative catalytic functional clusters (Fig. 1a). A fourth cluster was conformed with the 3 identified CKIN1 regulatory sequences aligned with their Arabidopsis, *Homo sapiens* and *Saccharomyces cerevisiae* orthologs (Fig. 1b). Although closely related to CKIN2, CKINL was excluded from the kinase group during alignments curation. The first identified catalytic cluster involved the SnRK1/AKIN complex, including Chlamydomonas catalytic subunit α, CKIN1, (Serin/Threonin Kinase (PTHR24343), UBA, and KA1/αCTD domains) and CKIN1L (Serin/Threonin Kinase (PTHR24343) and UBA domains). CKIN1L displayed also unique features as a long N-terminal unconserved sequence and lacked conserved Thr189, key into CKIN1 activation^41^, and the regulatory KA1/αCTD domain (Supplementary Figure S1). The second cluster, SnRK1 regulatory subunits, included those non-catalytic subunits of the SnRK1 complex: CKIN β (Immunoglobulin E-set/CBM and βCTD/ASC/AMKβI domains), CKIN βγ (Immunoglobulin E-set/CBM and CBS domains) and related CKIN γ (CBS domains). CKIN γ showed more identity to plant γ subunits and *Saccharomyces cerevisiae* γ-like SDS23^42^ than to true γ-acting proteins as plant βγ and human γ subunits (Fig. 1b). The sequences belonging to these two clusters were conserved across evolution as shown by its curated alignments using M-Coffee (Fig. 1c).

Sequences belonging to the Chlamydomonas CKIN2 subfamily were characterized by a SRK2 Serin/Threonin Kinase domain, which is shared with CKIN1. M-Coffee alignment tree arranged CKIN2 sequences into two clusters, CKIN2 (A) and CKIN2 (B) (Fig. 1a). The Serin/Threonin Kinase domain (PTHR24343) was present in all Chlamydomonas CKIN2 sequences and its Arabidopsis orthologs (Fig. 1c; Supplementary Figure S2; Supplementary Figure S3; Supplementary Table S3). Conversely, SRK variants SRK2C (PTHR24343:SF207), SRK2D (PTHR24343:SF169), SRK2E (PTHR24343:SF167), and SRK2F (PTHR24343:SF200) Serin/Threonin Kinase domains seem to be unique to Chlamydomonas CKIN2 subfamily. These domains are characteristic of the newly found elements (Supplementary Table S3). In plants, these kinases have two conserved serine or threonine phosphorylable residues into the activation loop required for gaining kinase activity^43^. Although Serin/Threonin Kinase domain showed slight variations across Chlamydomonas CKINs, it can be considered that activation region is conserved and functional since this site has been revealed as differentially phosphorylated in various environments (Supplementary Figures S2, S3) after reanalyzing available phosphoproteomic datasets^44–46^.

Chlamydomonas CKIN2 (A) has regions homologous to Arabidopsis osmotic stress-dependent activation domain I after its kinase domains, where CKIN2 (B) sequences showed a conserved region after kinase domain but less similar to the plant domain I (Supplementary Figures S2, S3). In CKIN2.2 and 2.6–2.8 ABA-dependent activation domains II-like sequences also followed domain I, but were smaller and less rich in acidic residues than its Arabidopsis counterparts^36^ (Supplementary Figure S2).

Most Chlamydomonas CKINs showed sequence variations not present in Arabidopsis SnRKs. Similarly to the extra loops previously described in the kinase domains of CKIN2.1, 2.3 and 2.4^36^ (Supplementary Figures S2, S3), extra sequences were found not only restricted to kinase domain. Some domains I and II, and N- and C-terminal regions showed length and sequence variation (Supplementary Figures S2, S3). These extra sequences harbored phosphorylation sites, glycine rich patches (into C-terminal tails) or in the case of CKIN2.4 and 2.14, coiled-coil predicted regions (Supplementary Figures S2, S3).

**Figure 2:**
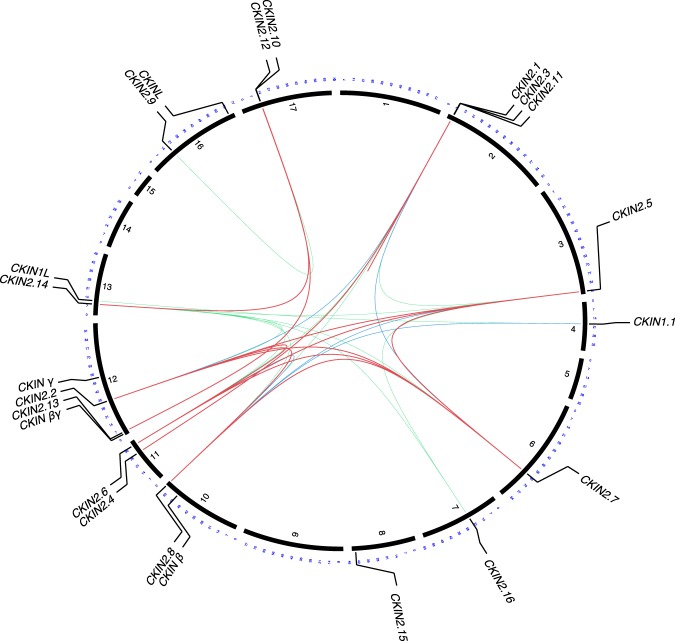
CKIN family evolution in Chlamydomonas. Chlamydomonas CKIN family genes were represented along chromosomes and gene duplications showed as links between duplicated elements. Link colour and thickness show BLASTP e-value and % identity based duplication confidence. Red thicker links joins genes coming from highly probable duplication events with e-values lower than 10^−50^ and more than 50% identity, blue links joins genes coming from mid probable duplication events with e-values lower than 10^−45^ and more than 45% identity and green links joins genes coming from low probable duplication events or ancient duplication events with e-values lower than 10^−40^ and more than 40% identity.

CKIN2 subfamily was further analyzed along CKIN1 and its regulatory subunits to determine gene duplicities and evolutive relations between the members of this family (Fig. 2). CKIN genes mapped to 12 chromosomes,  being *CKIN2*.*10* and *2*.*12*, and *CKIN2*.*1*, *2*.*3* and *2*.*11* close in their respective chromosomes. Homology results showed that *CKIN2*.*2* and *CKIN2*.*5–2*.*7* evolved by duplication of *CKIN2*.*8*, the sequence exhibiting higher homology to higher plants and closer to *CKIN1*. On the other hand, *CKIN2*.*1*, *2*.*3* and *2*.*4*, *CKIN2*.*14* and *2*.*10*, and *CKIN2*.*12* and *2*.*13* evolved from three different ancestors no longer conserved in Chlamydomonas.

Sequences belonging to Arabidopsis SnRK3 subfamily, characterized by the presence of a Ser/Thr kinase (PTHR24343), Ca-dependent protein kinase (PTHR24347), and NAF/FISL (IPR018451) domains, were not found in the Chlamydomonas genome (Supplementary Table S3).

**Figure 3:**
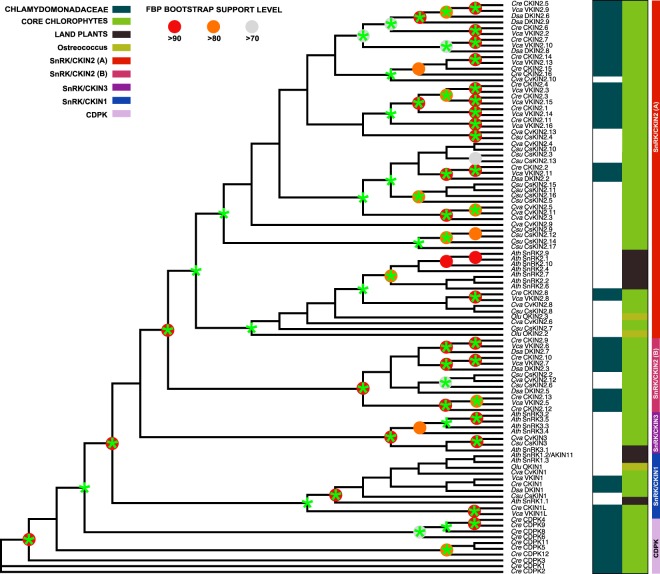
SnRK/CKIN family evolution between Chlorophyta and higher plants. TCS curated M-Coffee alignment based MS sequence tree. TBE bootstrap confidences over 80% are marked with a green asterisk over nodes. *Chlamydomonas reinhardtii* (*Cre*) SnRK sequences were included along with *Arabidopsis thaliana* (*Ath*), *Chlorella variabilis* (*Cva*), *Coccomyxa subellipsoidea* (*Csu*), *Dunaliella salina* (*Dsa*), *Ostreococcus lucimarinus* (*Olu*) and *Volvox carteri* (Vca) orthologues. Tree arranged SnRK/CKIN sequences into 4 clusters, namely SnRK/CKIN3, SnRK/CKIN1, SnRK/CKIN2 (A) and SnRK/CKIN2 (B). Subfamily distribution between species shows SnRK3 restricted to land plants and basal core Chlorophytes. SnRK/CKIN2 subfamily resulted highly variable between species, from the reduced and close to land plants Ostreococcus subfamily to the diverse and large Chlamydomonadaceae subfamilies.

The employment of the strategy described above over *Ostreococcus lucimarinus*, *Chlorella variabilis*, *Coccomyxa subellipsoidea*, *Volvox carteri*, and *Dunaliella salina*, showed divergent SnRK/CKIN family structures between land plants and microalgae (Supplementary Table S4). All studied species had one SnRK1 catalytic subunit, but had different elements belonging to SnRK2 and 3 subfamilies. Chlamydomonadaceae species lacked SnRK3 subfamily orthologs but had large and diverse SnRK2/CKIN2 (A) groups composed by CKIN2 proteins with uncharacterized insertions and long C-terminal extensions, and others similar to plant SnRK2s as it was observed in Chlamydomonas. Chlorella and Coccomyxa shared with Chlamydomonadaceae a SnRK2/CKIN2 (B) group and a reduced SnRK/CKIN2 (A) group making a SnRK2 subfamily more similar to Arabidopsis compared to Chlamydomonoceae. In turn, Chlorella and Coccomyxa had SnRK3 elements. Interestingly, Ostreococcus, the closer genome to land plants among the analyzed, contains only a small number of Arabidopsis-like sequences not including SnRK3, making the smallest SnRK families among the studied ones (Fig. 3, Supplementary Table S4).

Moreover, the use of iTAK database^47^ and kinase classification^48^ over all Chlamydomonas, Volvox, Dunaliella and Ostreococcus genomes showed similar grouping than proposed following our approach. SnRK1 sequences were classified as CAMK_AMPK and almost all found SnRK2 sequences fell into SNF like CAMK_OST1L kinase group. In Chlamydomonas, the divergent CKINL and CKIN1L sequences made an exception being classified respectively into CAMK_CAMKL-PASK-PIM and CAMK_Cr-1, close to SNF microalgae exclusive groups. Ortologs to these sequences were also found in Volvox, Dunaliella, and Chlorella.

### Expression profiles of Chlamydomonas CKINs under abiotic stress

RNA was isolated from CC-503 strain after 48 h of exposure to different stressful situations described in Table 2. The application of these stresses significantly affected cell growth, reducing multiplication rate in all cases, except for phosphorous limitation and UV irradiation (Supplementary Figure S4). These results were complemented with a targeted analysis of available RNAseq datasets studying the response of this organisms to nitrogen^49,50^, sulphur^37^, iron and carbon dioxide^51^ deficiencies and hydrogen peroxide-induced oxidative damages^52,53^.

**Table 2:** Variations of the basal HAP culture media to test different abiotic stress conditions *in Chlamydomonas* CKINs.

Assay	Modification of basal media
Nitrogen deprivation (-N)	Substitution of ammonium chloride by potassium chloride.
Carbon deprivation (-C)	Removal of carbon sources from the media.
Sulphur deprivation (-S)	Substitution of MgSO_4_, ZnSO_4_, FeSO_4_, and CuSO_4_ by MgCl_2_, ZnCl_2_, FeCl_2_, and CuCl_2_.
Phosphorous limitation (5% P)	Reduced phosphorous (5% of the TAP medium).
Heat Stress (40 °C)	Increase incubator temperature to 40 °C.
Cold Stress (4 °C)	Decrease incubator temperature to 4 °C.
UV radiation Stress (UV)	30 minute UV irradiation each 24 h.
Salt stress (0.25 M NaCl)	Addition of 0.25 M sodium chloride to basal media.
Osmotic stress (20% PEG)	Addition of 20% (w/v) PEG 4000 to basal media.

**Figure 4:**
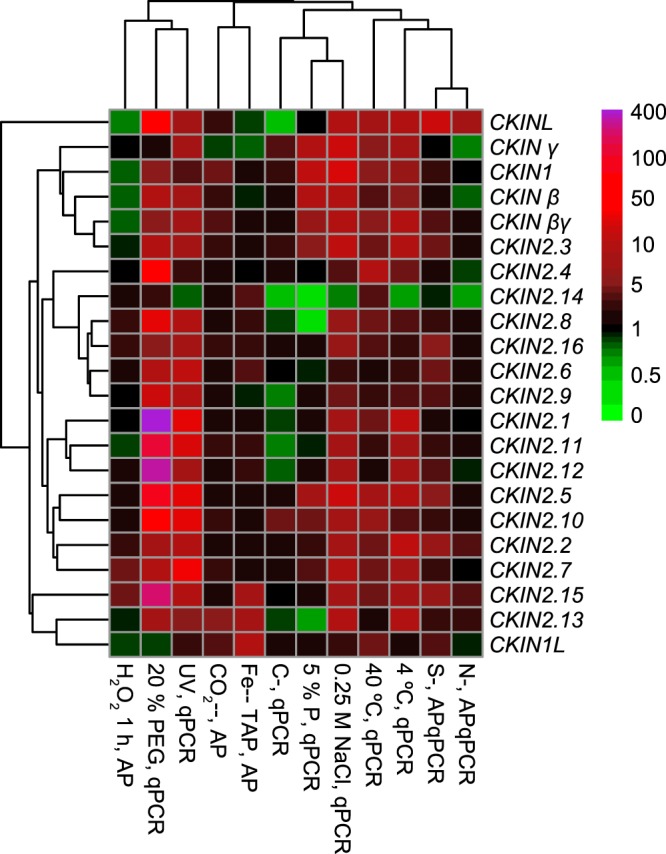
Heatmap and cluster representation of the changes in the abundance of CKIN family members under tested stress situations compared to control growth. Tree was built based on Euclidean distances and WPGMA aggregation method. Normalized fold-change values (z-scores) were used to avoid artefacts related to the different expression magnitudes of the members of this family. Legend: nitrogen (N-, APqPCR), carbon (C-, qPCR), and sulphur (S-, APqPCR) deprivation, phosphorous limitation (5% P, qPCR) deprivations, heat and cold stress (40 °C, qPCR and 4 °C, qPCR, respectively), UV radiation stress (UV, qPCR), salt (0.25 M NaCl, qPCR) and osmotic (20% PEG, qPCR). AP: expression data obtained from AlgaePath Database, qPCR: RT-qPCR dataset obtained in our laboratory.

The expression of Chlamydomonas CKIN genes greatly varied under the abiotic stresses tested, with different ranges of overexpression/repression in function of the analysed gene and stress (Fig. 4, Supplementary Table S5). PEG-induced osmotic and UV stresses triggered the strongest responses of this family, with an average CKIN abundance increase of 3.9- and 4.5-fold respectively and compared to control. Contrarily, the transition from mixotrophic to autotrophic conditions, H_2_O_2_-induced oxidative damage, and nitrogen limitation did not induce overall abundance change of the CKIN family genes. Although this reflects the dynamics of the entire CKIN family under several stresses, it is more interesting to analyse the dynamics of specific CKIN genes to look for possible targets for future bioengineering studies.

CKIN genes also showed different ranges of response within each treatment, from the small abundance variations of *CKIN2*.*14* and *CKIN1L* (2.28- to −10-fold and 2.94- to −2.43-fold change; maximum and minimum change considering all stresses, respectively) to the strongest response of *CKIN2*.*1* and *CKIN2*.*12* (440- to −1.47-fold and 354- to −1.28-fold change respectively). Salt, temperature, UV and PEG-induced osmotic stress caused the overexpression of all genes of these family at different levels, except for *CKIN2*.*14*, downregulated under salt, low-temperature and UV stress and *CKIN1L*, downregulated by PEG. On the other hand, carbon limitation (both the reduction of available CO_2_ and the transition from mixotrophic to autotrophic growth) led to the overexpression of genes belonging to the CKIN1 complex, *CKIN2*.*3*, *CKIN2*.*10* and *CKIN1L*, while nutrient deficiencies (S, N, P, Fe) induced gene-specific responses. Although Arabidopsis γ and AKIN10/11 or SnRK1.3 interaction have not been identified, Chlamydomonas CKIN γ (close to plant γ and Saccharomyces SDS23) showed a close expression pattern to CKIN1 and its regulatory subunits clustering in the same group. Moreover, *CKINL* differential expression pattern under tested stresses supported its exclusion from the CKIN2 subfamily.

### Changes in CKIN expression in response to ABA

**Figure 5:**
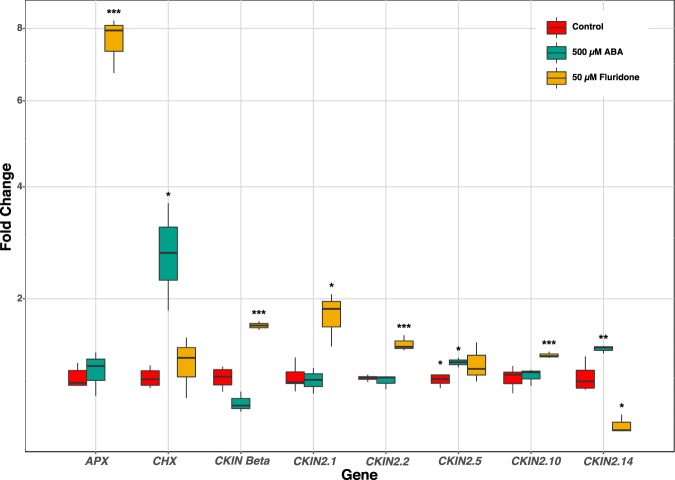
Expression change of CKINs after 48 h of culture in 500 µM ABA or 50 µM fluridone. Carotene hydroxylase (*CHX*), an ABA inducible gene, and Ascorbate peroxidase (*APX*), an oxidative stress inducible gene, were included in the analysis to test the effectiveness of the treatment. This plot only shows the 6 CKINs that were responsive to either ABA or fluridone treatment (ANOVA, *p < 0.05, **p < 0.01, ***p < 0.001, n = 6). RT-qPCR data was processed with EasyqPCR package according to Hellemans *et al*. (Hellemans *et al*., 2004).

Due to the importance of ABA in SnRK signalling in other biological systems we tested the expression levels of all CKIN genes after 48 h of culture in basal media supplemented with 500 µM ABA following Yoshida *et al*.^38^ or 50 µM fluridone. The expression of 6 out of 22 CKIN genes were affected under one or both culture conditions (Fig. 5). *CKIN2*.*14* was upregulated under ABA treatment but downregulated with fluridone. *β subunit*, *CKIN2*.*1*, *2*.*2*, and *2*.*10* were upregulated with fluridone, whereas only ABA addition upregulated *CKIN2*.*5*.

### STRING and ChlamyNet based CKINs interaction and co-expression networks

**Figure 6:**
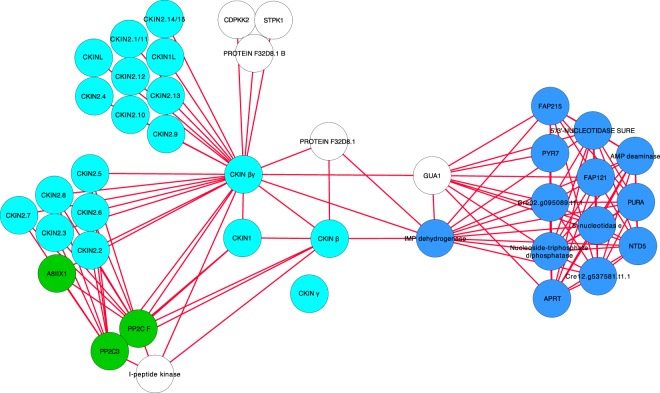
High confidence interaction network of CKIN proteins. CKINs (light blue) interact between them and with major regulatory groups according to KEGG classification: hormone-signalling response (green), nitrogen acquisition and derived pathways (dark blue). White nodes represent bridge proteins.

CKINs showed to be a part of a complex interaction network, as shown into the STRING^54^ and ChlamyNet^55^ databases. STRING encompasses a collection of predicted and experimentally proven protein-protein interactions in Chlamydomonas and other species. The Chlamydomonas CKINs present in the STRING database turned out to be associated between them and with several biochemical and regulatory pathways (Fig. 6; Supplementary Figure S5). Three main CKIN clusters were defined based on the interactions observed. The first one, the hub of this network, includes CKIN α, β, and βγ subunits of the CKIN1 complex. This cluster is directly linked to CKIN2, carbon and nitrogen metabolism, and mRNA splicing. In addition, it was indirectly linked through Ca-dependent mechanisms to autophagy and DNA remodelling and maintenance mechanisms. No interactions were found for the non-interacting CKIN γ subunit. Second and third CKIN clusters comprise CKIN2 subfamily, being linked to different Protein Phosphatase 2 C (PP2C) family phosphatases. CKIN2.4, 2.9, 2.13, 2.16, and CKINL only showed interaction with PP2CF, PP2C3, and A8IIX1 while the other set of CKIN2 and CKIN1L was also interacting with 9 other PP2C and related phosphatases. ChlamyNet database comprises a Chlamydomonas transcript correlation network. Eight CKINs were identified in this database being part of several clusters (Supplementary Figure S6). CKIN β, CKIN2.12, and CKIN1L were into the same group, directly connected to carbon (macromolecule and hexose metabolism, photosynthesis) and nitrogen metabolism (amino acid metabolism and protein turnover). CKIN2.6 formed a second cluster directly related with autophagy elements. CKIN γ conformed the third major cluster interacting with transcriptional regulation elements, autophagy and protein folding/assemblage. CKIN 2.3 and 2.16 were also related to transcriptional regulation and CKIN 2.7 to autophagy, heat response and protein folding.

Other proteomic and metabolomic interaction networks, as the Chlamydomonas nitrogen starvation and recovery response STRING based network presented by Roustan *et al*.^56^, and the nitrogen starvation response sPLS correlation network published by Valledor *et al*.^5^, were surveyed for CKIN or CKIN related elements. Although no CKINs were identified into the first network, interactants as the flagellar associated protein (FAP121) and the protein kinase CDPKK2 were shared between CKIN and nitrogen stress response network. The second network only included CKIN γ.

## Discussion

The SnRK family is conserved in all eukaryotes, as SnRK1/SNF1/AMPK/CKIN1, functioning as a sensor of cell energetic status^18,57,58^. In plants, this family evolved into two more subfamilies, 2 and 3, which are key players in ABA-dependent and -independent stress response mechanisms^25,31,59^. In Chlamydomonas, SnRK/CKINs were initially described under sulphur deprivation^60^, and later related to a wide range of stress responses^5,35–37^ and indicated as potential targets for increasing the production of industry-demanding biomolecules^32^. However, these studies focused on the functional characterization of some CKINs rather than defining the whole family, explaining why only 10 CKINs were described in Chlamydomonas so far, when in land plants like Arabidopsis this family comprises 38 members^27^.

Mining Chlamydomonas genome using homology searches allowed the definition of a large number of candidate genes, most of them only homologous to the Ser/Thr kinase domain. Furthermore, CKIN and CDPK protein families showed great similarity, being the absence of a Ca-binding EF-hands domain the key to distinguish these families^27^. In consequence, all BLAST hits were later curated by protein domain analyses. This strategy was later expanded to a domain-based genome mining looking for sequences containing all the domains required for a protein to be classified as CKIN. This proved to be useful allowing the incorporation of two novel CKIN sequences (*CKIN2*.*12*, and *CKIN2*.*15*) otherwise lost during data mining.

Overall, in silico genomic analyses resulted in the characterization of 21 proteins belonging to the CKIN family. As this family has not been deeply studied in other algae, comparisons are difficult to perform but recently, one of these analyses described only 2 elements belonging to SnRK1/CKIN1 subfamily in *Volvox carteri*^61^. Out of these, only one sequence can be undoubtedly classified as SnRK1, since its kinase, UBA, and KA1/αCTD domains are highly conserved across evolution^62^. The other sequence cannot be assigned to this group since regulatory residues were not present, being more similar to CKIN1L. Conversely, any SnRK2/CKIN2 elements were described in this species. The application of the described above workflow to the analysis of Volvox, Dunaliella, Coccomyxa, Chlorella and Ostreococcus genomes allowed the definition of progressively larger and more complex SnRK2s subfamilies as the species were closer to Chlamydomonas. Identified SnRK kinase members in Chlamydomonas, Volvox, Dunaliella and Ostreococcus were identified as such into the iTAK database^47^ supporting Chlamydomonas CKINL exclusion. These analyses validated the method capabilities to discover new SnRK/CKINs, highlighting the need of specific methodologies beyond homology searches, such as protein domain analyses, for the fully characterization of a protein family when dealing with families showing atypical evolution such as CKIN2 subfamily in algae^63^. Interestingly, from the genomes analysed, only land plants and humans had more than one SnRK1. SnRK1/AMPK duplication is common between plants and vertebrates whereas no more than one Snf/CKIN1 subfamily member has been identified in fungi and invertebrates^21^. This suggests a probable complexity related feature. Vertebrates and plants needed to diversify this regulatory mechanism to cope with their more complex life cycles and somatic structures, or more stringent environments^64,65^. This increasing somatic/environmental complexity fits the functional diversification found in some land plants and vertebrates SnRK1/AMPKs. Supporting this is the Arabidopsis SnRK1.3 reproductive tissues focused expression^66^, same species AKIN10/11 differentiated response under stress^64^ and muscle/liver focused expression of human AMPK α2 which is related to osteogenesis and adipogenesis^67^.

On the other hand, and despite the conservation of SRK2 Serin/Threonin kinase domain of the CKIN2 subfamily, more efforts are needed to be paid to unequivocally classify these elements. If CKIN2 subfamily originated from duplication and divergence of CKIN1, a common ancestor or “founder like” to all SnRK2/CKIN2s would diverse this subfamily in different groups ranging from CKIN1 to land plants SnRK2s. Both CKIN2 subgroups (A/B) were conserved within core Chlorophytes, while those closer to SnRK1, CKIN2 (B), were lost or changed in land plants. SnRK2/CKIN2 (A), including plant SnRKs, was larger and more diverse in core Chlorophytes, with almost all this sequence diversity being Chlamydomonadaceae exclusive. In Chlamydomonas, CKIN2.2 and 2.8 can be considered the common ancestors of most of its CKIN2 (A) sequences, despite some ancestor of this group long CKIN2s may be lost along CKIN2 (B) group ancestor. Furthermore, despite being present in Chlorella and other Chlorophytes, this ancestor CKIN2 (B) group has been lost in Ostreococcus, exhibiting only plant-like CKIN2s, this may also be happening due to its reduced genome size. Although atypical and different from land plants, algal CKINs are supposed to be engaged in most of the functions land plants SnRKs are involved. This is sustained by the gene balance hypothesis, which explains the conservation observed, predicting that transcription factors and proteins belonging to signalling networks are more likely to be retained^68^.

In plants, SnRK1-based sugar signalling acts as a master regulator of carbon/energy ratios and is strongly linked to hormonal regulation and different signal transduction pathways^69,70^. In Chlamydomonas this complex resulted to be very sensitive to most of the imposed stresses. However, the suppression of carbon in culture media did not caused the highest overexpression rates of this complex, being more responsive to salt and extreme temperatures. The damages that these stresses represent to algae causing an energy shock are probably greater than those from removing acetate from media (that will still interchange CO_2_ with air). The metabolic remodeling required to survive under extreme conditions mediated by SnRKs has been reported in different plant systems^35,71^, being the interaction of the SnRK1 complex with specific transcription factors and kinases a basic mechanism to trigger proteome and metabolome remodelling. CDPKK2, a Chlamydomonas ortholog to Arabidopsis GRIK1, known for activating the land plant SnRK1^72^ is one of the kinase intermediaries highlighted into presented STRING based SnRK network. The kinase was also present as central node into Chlamydomonas nitrogen depletion and recovery stress network^56^ supporting the complex activity under abiotic stresses other than carbon depletion.

Conversely to CKIN1, CKIN2 proteins were more difficult to classify due to its partial divergence to its land plant counterparts. Previously described CKIN2 elements, falling all into the CKIN2 (A) group, exhibited a high similarity to its Arabidopsis SnRK2s orthologs. On the contrary, newly discovered elements, all CKIN2 (B) sequences and the Chlamydomonadaceae exclusive SnRK2 (A) sequences, were more dissimilar. However, all Chlamydomonas CKINs exhibited a Ser/Thr kinase domain and, in the case of CKIN2 (A) sequences a land plant-like regulatory domain I which is less conserved in Chlamydomonadaceae sequences CKIN2.11 and CKIN2.15–16. This domain is related to osmotic and salt stress responses in plants^26,63^. The domain I function seems to be mostly conserved in Chlamydomonas, as all elements containing it, except CKIN2.14, were overexpressed under hyperosmotic and saline stresses. Interestingly CKIN2 (B) group sequences exhibited a conserved region after kinase domain which is different than land plants domain I. CKIN2 (B) sequences and CKIN2 (A) CKIN2.11 and CKIN2.15 were also overexpressed under hyperosmotic and saline stresses probably due to the existence of an algae specific domain and other sensing/signalling mechanisms respectively.

The presence of different regulatory sequences, some of them elongated, may explain the differential classification of CKIN2 after M-Coffee analysis, which focused on most conserved regions. CKIN2 (A) grouped the closest sequences to the common land plant-algae ancestor and all Chlamydomonadaceae exclusive ones. Some of these sequences were almost identical in length and sequence to Arabidopsis while most, including the closest Chlamydomonas CKIN2 to Arabidopsis SnRK2s (CKIN2.8), had long C-terminal ends and/or extra sequence loops. These features are probably related to a multiple environmental stress response capacity. CKIN2 (A) sequences have a heterogeneous response pattern under different stresses. Besides this, their expression levels under sulphur or nitrogen starvation, phosphorous limitation and oxidative stress were higher than CKIN2 (B) sequences.

CKIN2 (B) contained core Chlorophyte exclusive sequences. Sequences of this smaller group are more homogeneous, with a conserved region after kinase domain in place of regulatory domain I and long C-terminal ends (excepting CKIN2.9). Within the CKIN2 (B) cluster, CKIN2.13 and 2.10 were the most overexpressed CKIN2 under carbon starvation and autotrophic growth respectively, surpassing CKIN1 complex. This correlates this CKIN2 (B) “ancestor” group sequences with an energy stress related function commonly associated to CKIN1 complex.

Besides differences, CKIN2 (A/B) sequences share the osmotic/UV responsiveness, excepting CKIN2.14 and 2.4, CKIN1L, and CKIN γ. CKIN2s as *CKIN2*.*1*, *2*.*12*, and *2*.*15* were strikingly upregulated (c.a. 400-fold) under osmotic stress.

Response heterogeneity and group size makes difficult to propose a common regulatory mechanism for CKIN2 (A). Moreover, despite they share with CKIN2 (B) and land plants a well conserved kinase-dependent activation loop^43,73^, most of its sequences also display extra sequence loops/elongations whose function is probably diverse. Furthermore, the presence of phosphorylation sites, coiled-coil protein interaction motifs, glycine and glutamine rich regions in CKIN2 (A) sequences points to a probable CKIN2 regulatory function. Plant SnRK2 protein ends usually have an important role in their regulation trough phosphorylation^74^. Moreover, coiled-coil structures, know by their function in protein-protein interactions^75^, hold different kind of protein interactions in plant SnRKs, such as the interaction with PP2Cs^76^. In addition to the activation loop and these C-terminal potential regulatory regions, the regulation of SnRK2 in Arabidopsis requires the presence of two regulatory domains. All CKIN2 (A) had a domain I with different degrees of degeneration, but domain II like regulatory sequences could only be recognized into CKIN2.2 and CKIN2.6–2.8, the closest CKIN2s to land plants SnRK2 subfamily.Although regulatory domain II is characteristic of some CKIN2 (B), these sequences are shorter that those present in land plants. In land plants this domain, with its characteristic acidic patch, is present in highly ABA responsive elements, being named ABA box, despite some SnRK2s can respond to ABA without this structure^26,63,77^.

Interestingly, *CKIN2*.*14* and *CKIN2*.*5* were the only responsive elements to exogenous ABA, regardless of not having acidic patches in their sequences. ABA regulatory mechanisms are well described in land plants, where the acidic domain II mediates the inactivation of the kinase through a SnRK2-PP2C interaction^74^. ABA, interacting with PYR /PYL/RCAR ABA receptors inhibits PP2C, allowing the kinase activation^78,79^. Although all of these elements are not present in microalgae or have a very low similarity to higher plants^39,40^, string-based protein-protein analyses demonstrated the interaction between SnRK2s and PP2C, protein phosphatase Mg2+/Mn2+ dependent (PPM) and Protein phosphatase type 1 isoform K (PP1K) phosphatases, and CAB and CDP kinases, all core players in ABA signalling^80^. Therefore, *CKIN2*.*14* and *2*.*5* might be elements in an ABA regulatory pathways in Chlamydomonas, highlighting the need of novel SnRK-PP2C interaction sequences and alternative ways in which ABA breaks this interaction. This hypothesis is also supported by the effect of exogenous application of fluridone, which increased the expression of *CKIN2*.*1*, *2*.*2*, *2*.*5* and *2*.*10* despite it can also be considered as an oxidative stress induction since CKIN *β* subunit was also induced.

Results clearly showed low ABA signalling dependence of CKIN2s in Chlamydomonas. In lesser extent, ABA-independent regulation of SnRKs occurs in land plants, as in the case of Arabidopsis *SnRK2*.*1*, *2*.*4*, *2*.*5*, *2*.*9* and *2*.*10*, induced by osmotic stress following an ABA-independent pathway^63^. Arabidopsis ABA-independent SnRKs regulate transcripts of stress related genes under hyperosmotic conditions, thus complementing ABA-dependent SnRK2s function^81^. In Chlamydomonas, ABA-independent CKIN2s were also responsive to osmotic stress, as in land plants. But contrary to plants, specific responses to low nitrogen^5^ or sulfur deprivation^36^ seem to be triggered in an ABA-independent manner. These results support the idea that the plant-specific SnRK2/CKIN2 subfamily plays a crucial role in stress response signaling both in Arabidopsis and Chlamydomonas.

These mechanisms are not entirely related to energy-saving decisions, but lead to a complex remodelling of cell metabolism, as demonstrated by the interactions with DNA repair and maintenance pathways and TOR in Arabidopsis^82^, and in Chlamydomonas to a similar and complex interaction network as it was proposed by STRING and ChlamyNet analyses. The fact that the expression of a large number of CKIN2s is induced by a single stress suggests a great compensatory effect or pleiotropy within this family in Chlamydomonas. It is well-known that the consequence of most stresses is oxidative damage and/or low energy syndrome^83,84^ but it is striking the high number of elements triggered in Chlamydomonas under most of the studied stresses given their low intensity. On the other hand, the high responsiveness to osmotic, salt, and UV stresses of this family is crucial for a freshwater alga in natural environments, since they cannot control neither water quality nor environmental UV irradiation. Thus, a fast, flexible, and efficient mechanism is required to ensure algae survival under unfavourable environmental conditions.

The apparent lack of specificity of CKIN confronts to other plant systems, with elements with very specific functions and stress responsiveness. Land plants also had a third SnRK subfamily, comprising proteins kinases interacting with calcineurin B-like calcium binding domains^63,85^, mostly involved in drought and salt resistance, being the SOS (salt overly sensitive) the best-known mechanism^86^. The characteristic NAF/FISL^27^ domain of SnRK3 is not present in Chlamydomonas, but Chlorella and Coccomyxa have one element. This suggests that SnRK3 is not only characteristic of land plants but is also present in the last common ancestor and lost in some core Chlorophytes. Homologs of this sequence were not found in Ostreococcus (the closest microalgae to land plants) but this would be easily explained by its reduced genome. Moreover the reduced SnRK3 subfamily size in microalgae makes easy their lost through mutation, being probably compensated in their function by SnRK2s, specific CDPK proteins^87^ or by SNF/SKP1/Ubiquitin ligase complexes, already identified as key elements of hormone, sugar, and stress responses^88^. In line with this, Chlamydomonas, Volvox, and Dunaliella have larger and more diverse SnRK2/CKIN2 subfamilies than Chlorella and Coccomyxa, both containing one SnRK3 element.

The genome-wide approach used in this work over Chlamydomonas for the identification of its CKIN family have completed previous work in this species while extending it to other microalgae. This kinase family description has shed light to its unique structure and sequence features in Chlamydomonadaceae, highlighting the conserved abiotic stress sensitivity of the Chlamydomonas members. Thus, paving the way for the description of novel CKIN and probably ABA mediated stress responsive pathways in microalgae. The shared and unique core Chlorophytes SnRK/CKIN family structure make Chlamydomonas a suitable system for novel stress response mechanisms identification and a better fitted model than land plants for the identification of algae specific targets for biofuels and secondary metabolites production enhancement in these species.

## Methods

### CKIN sequence identification and classification in Chlamydomonas

Chlamydomonas CKIN family genes were initially defined by BLASTP comparison against the Chlamydomonas proteome v5.5^89^ available at Phytozome^33^ employing Chlamydomonas^35,36^ and Arabidopsis^25^ previously identified CKINs and SnRKs as query (Supplementary Table S1). Homology was considered for e-values lower than 10^−25^ generating a first uncurated sequence list (Supplementary Table S2).

On a second step, Inter Pro Scan^90^ was used to define the domain structure of all candidate sequences, filtering out those proteins with no SnRK domain structure (e.g. CDPKs). Furthermore, BLASP query sequences domains (Supplementary Table S3) were used as reference to search for potential CKINs into the Chlamydomonas genome using BIOMART^91^. Protein sequences with characteristic SnRK/CKIN domains, were aligned with M-Coffee^92^.

Maximum likelihood (ML) sequence trees were built into PhyML^93^ platform employing M-Coffee general alignment distances after a TCS (Transitive Consistency Score)^94^ alignment filtration. One hundred bootstrap replicates were done into the same PhyML platform over the filtered alignment data to assess tree consistency. Different tree topologies were evaluated including a different set of ortholog SnRK proteins sequences (Supplementary Table S6) and Chlamydomonas CDPK sequences. Same tree-group sequences were aligned together using M-Coffee to validate sequence adhesion to their group through distinctive sequence motif conservation. These block alignments were curated using g-blocks^95^. COILS^96^ and different phosphoproteomic datasets were used for alignment enrichment with coiled-coil predicted regions and phosphosites.

Gene duplication was inferred from tree topologies and intra SnRK sequence comparison by BLASTP^97^. Intra tree group comparisons of BLASTP e-values and identity % were used as parameters for duplicity consideration. Three confidence thresholds for high, medium and low duplication origin probability were respectively defined at 10^−50^, 10^−45^, and 10^−40^ for e-values, and 50, 45, and 40 for identity %.

Volvox, Dunaliella, Coccomyxa, Chlorella and Ostreococcus sequences homologous to Chlamydomonas and Arabidopsis CDPKs and SnRKs were obtained from Phytozome^98–101^ employing the methods previously applied to Chlamydomonas genome mining as described above. SnRK/CDPK sequence groups found in Chlamydomonas, Volvox, Dunaliella *and* Ostreococcus were curated both manually and through iTAK application^47^. Identified SnRK/CKIN sequences in microalgae species and Arabidopsis along with Chlamydomonas CDPK as an outgroup, were aligned using M-Coffee. A ML sequence tree was built into PhyML platform from the TCS filtered M-Coffee global alignment distances. One hundred bootstrap replicates were done into the same PhyML platform over the filtered alignment data to assess tree consistency. Transfer bootstrap expectation (TBE) bootstrapping method^102^ with 100 replicates was used along conventional Felsenstein’s method for improving deep branches bootstrap support.

### Chlamydomonas culture and stress response characterization

Chlamydomonas strain CC-503 cells were grown on a closed incubator (25 °C, 120 rpm, 16 h light:8 h dark photoperiod and 190–200 µE m^2^ s^−1^ light intensity provided by warm white LEDs) in liquid HAP culture media^103^ supplemented with 10 mM sodium acetate at an initial cell density of 3–5 × 10^5^ cells mL^−1^. Basal media composition and growth conditions were changed according to Table 2 to test different abiotic stresses.

Fifty mL culture samples were collected at the beginning of the experiment (0 h) and 48 h after the start of the assay. Samples were centrifuged (4000 rpm, 6 min) and resulting cell pellet masses were estimated gravimetrically and frozen using liquid nitrogen. Four biological replicates of each stressful scenario were performed.

### Expression analysis of CKIN genes under a variety of environmental stresses

A precise estimation of SnRK abundance based on RT-qPCR was performed over 9 experimental situations (Table 2). RNA was extracted from the previously frozen pellets using the method described by Valledor *et al*.^104^. 1.7 µg of DNA free RNA was used for cDNA synthesis.

RT-qPCR analysis was performed using the CFX96 Touch Real-Time PCR detection system (Bio-Rad). The individual reactions contained 1x Maxima SYBR Green qPCR Master Mix (Thermo Scientific), 0.5 µM of each primer, 2% DMSO, and 0.7–0.8 µg of cDNA. The amplification protocol consisted in 1 × [95 °C, 10 min], 50 × [95 °C, 15 s; 61 °C, 30 s; Fluorescence reading] and a final melting curve. Relative expression levels were determined in 22 independent experiments for each primer pair (Supplementary Table S7). Each individual experiment was performed with two analytical replicates for each biological replicate from each condition tested. *UBQ* and *RCK1* were selected as endogenous controls after testing the expression stability of *IDA5*, *UBQ*, *TUB*, and *RCK1* with the geNorm software^105^. Gene expression of each SnRK was evaluated by calculating ΔΔCq values (Supplementary Table S5b) according to the recommendations proposed by Hellemans *et al*.^106^.

NGS-based transcriptomic datasets of *Chlamydomonas reinhardtii* available at the AlgaePath repository^107^ were mined to perform a complementary characterization of the CKIN family expression under stressful conditions. Five different datasets were analysed: sulphur depletion^37^, nitrogen deprivation^50^, low CO_2_ content^51^, oxidative stress and iron deprivation^53^. Each CKIN gene was searched using their gene accession, and its fold change variation under stress was obtained by comparison to non-stressed abundance values. In all cases, abundances within each dataset were normalized against its corresponding abundance in controls (Supplementary Table S5a). The integration of NGS and RT-qPCR abundances was done based on the z-score of the fold change variation between control and stress situation.

### ABA-induced regulation of CKINs

HAP medium was supplemented either with 500 µM ABA or 50 µM fluridone (carotenoid and ABA synthesis inhibitor through Phytoene desaturase inhibition) following previous works^38,108^. CC503 cell cultures were grown under the previously described basal conditions for 48 h, and compared to its corresponding controls. RNA extraction and RT-qPCRs were performed over 3 biological replicates each treatment. The effect of ABA and fluridone was monitored by quantifying the expression of the ABA-inducible genes Beta-Carotene Hydroxylase (*CHX*) and Ascorbate peroxidase (*APX*)^109^.

### Bioinformatic and statistical analyses

All the procedures for the identification and classification of Chlamydomonas SnRK were performed locally employing the bioinformatics suite Geneious v7 (Biomatters Inc.), with the exception of Inter Pro Scan^90^ and BIOMART^91^ searches that were performed at the European Bioinformatics Institute (ebi.ac.uk) and Phytozome (phytozome.jgi.doe.gov) websites, respectively.

Protein-protein functional interactions were identified by using STRING v10^54^ and ChlamyNet^55^ databases. CKINs protein sequences were uploaded to STRING application and database was queried considering Chlamydomonas as a reference organism. Two related networks were made, one showing highest confidence (over 0.85 STRING interaction score) known interactions. The other network including also high confidence (over 0.7 STRING interaction score) known and predicted interactions. CKINs protein sequences were also uploaded to ChlamyNet application, containing a Chlamydomonas transcript-based correlation network. A smaller network was made out of the original containing only first level CKIN interactions. STRING and ChlamyNET resulting networks were represented using Cytoscape v3.4 (Cytoscape Consortium 2016).

R v.2.4 software (R Core Team 2016) core functions and the gplots2 and pheatmap packages were used under the R Studio Environment (RStudio Team 2016) to perform the statistical analyses and heatmap plotting of transcriptomic data. ANOVA and t-test (α = 0.05 in both cases) were respectively employed for comparing the expression values (ΔΔCq) of CKINs under different stresses, values previously processed using EasyqPCR package for R^110^. When mining available datasets in Algaepath, the abundance of each CKIN under the different stress situations was compared to its corresponding non-stressed controls considering fold change.

## Electronic supplementary material

Supplementary Figures and tables

## Data Availability

Sequence data analysed in this work, with their IDs listed at Supplementary Table S4 and Supplementary Table S6 are available at phytozome, JGI Chlorella NC64A genome portal and NCBI nucleotide repositories; https://phytozome.jgi.doe.gov, https://genome.jgi.doe.gov/ChlNC64A_1 and https://www.ncbi.nlm.nih.gov respectively. RT-qPCR datasets generated and analysed during the current study are available from the corresponding author on reasonable request. Phosphoproteomic and transcriptomic datasets analysed during this study are included in Wang, H. *et al*. 2014, Werth, E.G. *et al*. 2017 and de la Fuente Van Bentem, S. *et al*. 2008 works^44–46^ (and its respective Supplementary Information files) as well as into the Algaepath and ChlamyNet databases at http://algaepath.itps.ncku.edu.tw and http://viridiplantae.ibvf.csic.es respectively.
