# Supplementary material for: Genome-wide identification and characterization of CKIN/SnRK gene family in Chlamydomonas reinhardtii
Source: Sci Rep. 2019 Jan 23;9:350. doi: 10.1038/s41598-018-35625-8 (PMC6344539; doi:10.1038/s41598-018-35625-8)
Supplement: Supplementary file 1 — Supplementary Figures and tables [file 41598_2018_35625_MOESM1_ESM.pdf]

Genome-wide identification and characterization of CKIN/SnRK gene family in *Chlamydomonas reinhardtii*

Francisco Colina[1], Joana Amaral[2], María Carbó[1], Gloria Pinto[2], Amadeu Soares[2], María Jesús Cañal[1], Luis Valledor[1,2,*]

[1]*Plant Physiology, Department of Organisms and Systems Biology and University Institute of biotechnology (IUBA), University of Oviedo, Oviedo, Spain;* [2]*Department of Biology and CESAM, University of Aveiro, Aveiro, Portugal.*

[*]**Corresponding author:** valledorluis@uniovi.es

Supplementary figures & tables

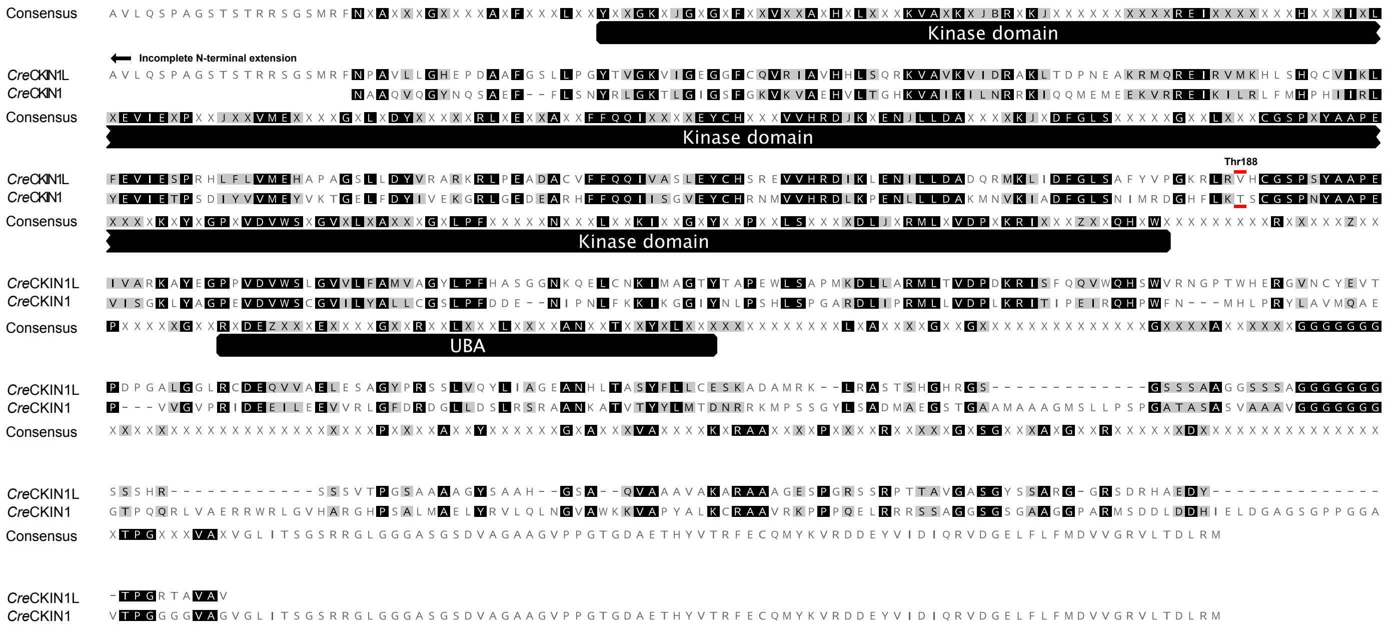

Supplementary Figure S1: Muscle based sequences structural analysis of *Chlamydomonas reinhardtii* (*Cre*) CKIN1 and CKIN1L. Both sequences share a highly analogous kinase domain followed by a shared UBA domain. CKIN1L has a long uncharacterized N-terminal region (not shown) that does not align with SnRK1' (←).

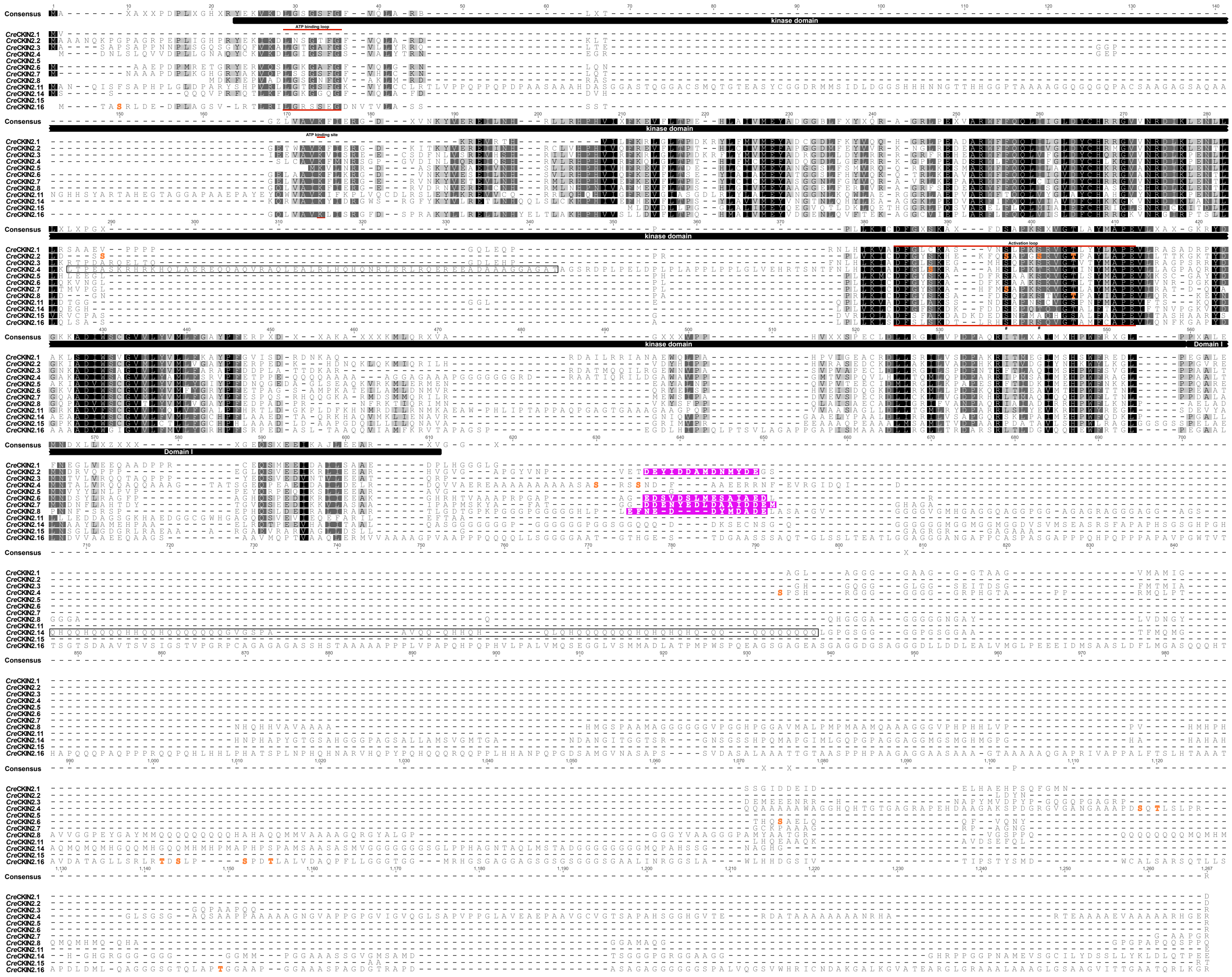

Supplementary Figure S2: M-Coffee based sequence structural analysis of Chlamydomonas (*Cre*) CKIN2 (A) group. CKIN2.4 and 2.11 show extra sequence loops and along CKIN2.16 long C-terminal ends. CKIN2.15 have a short N-terminal region. CKIN2.4 and 2.14 share predicted coiled-coil regions (rectangled) placed in CKIN2.4 into kinase domain inclusions and in CKIN2.14 in their C-terminal tail. Conserved ATP binding loop/site, and activation loop are marked. Within activation loop, conserved Serin/Threonin activation residues are highlighted (#). Phosphorylated S or T sites are marked in bold orange characters. Domain II like acidic patches into CKIN2.2 and CKIN2.6-8 are highlighted in pink.

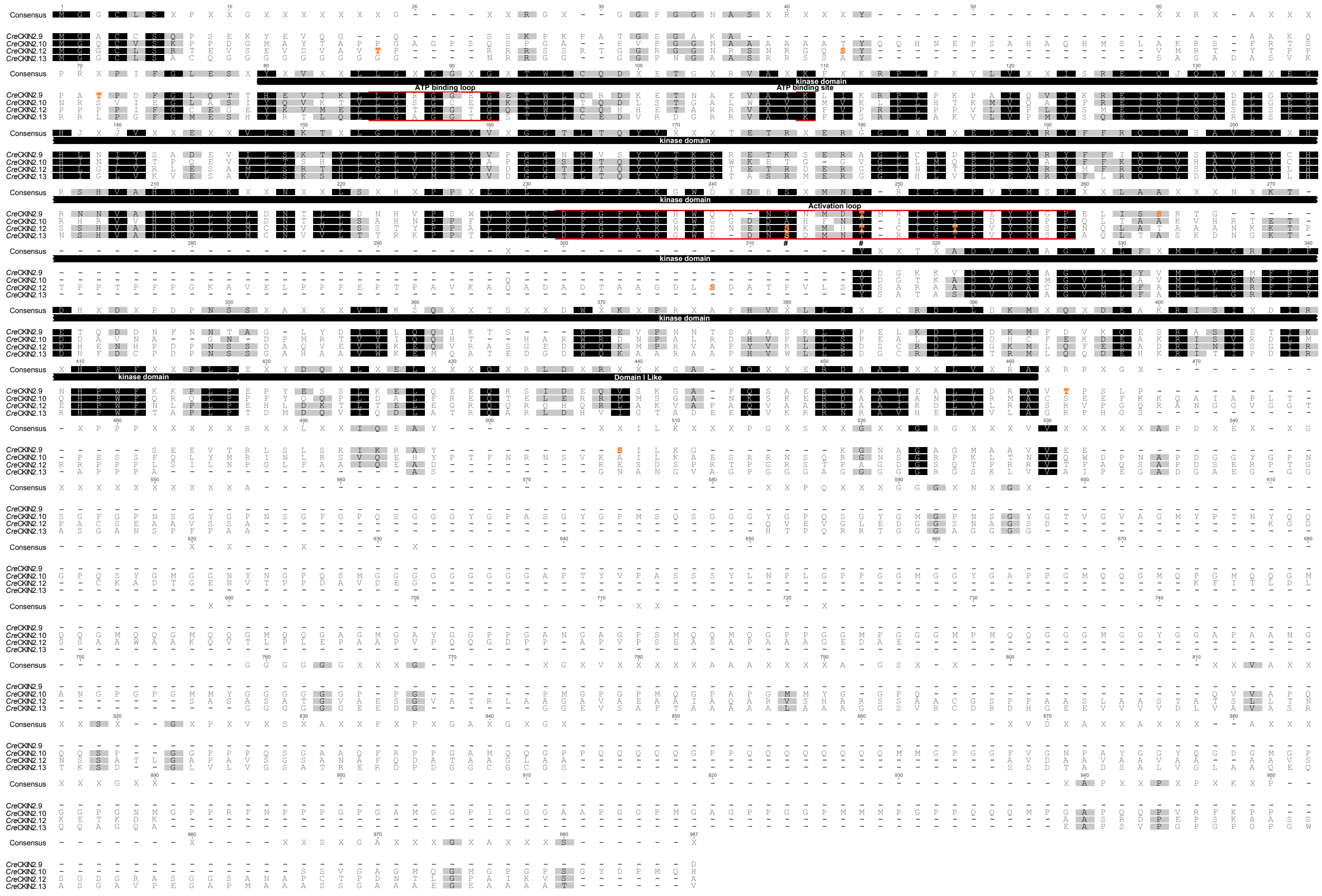

Supplementary Figure S3: M-Coffee based sequence structural analysis of Chlamydomonas CKIN2 (B) group. All sequences shared kinase domain sequence and a conserved structure (Domain I like) after the catalytic domain. Conserved ATP binding loop/site, and activation loop are marked. Within activation loop, conserved Serin/Threonin activation residues are highlighted (#). Phosphorylated S or T sites are marked in bold orange characters. These terminal regions were also rich in glycine residues in most sequences.

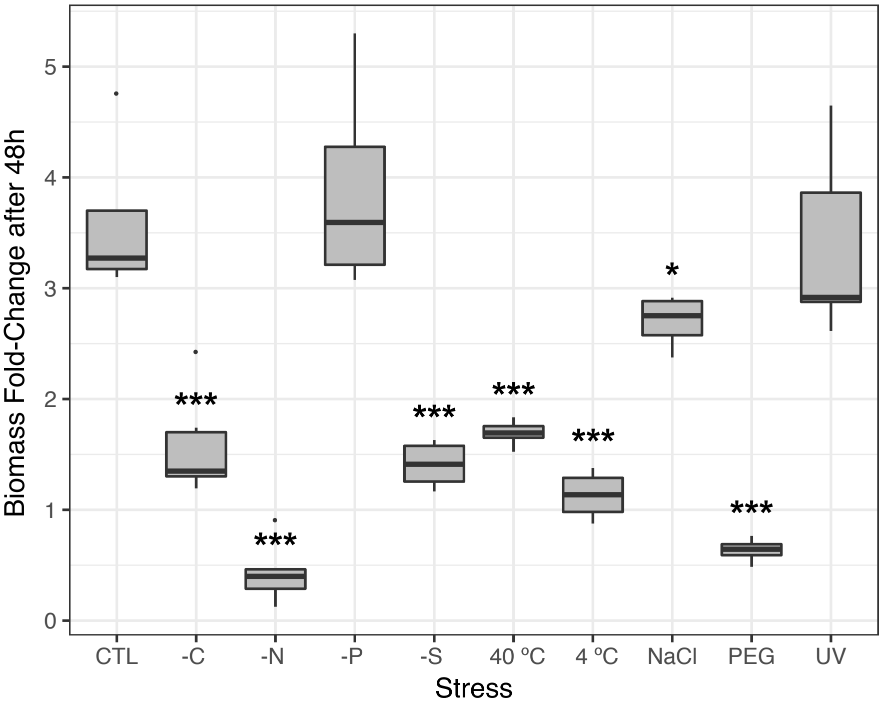

Supplementary Figure S4: Boxplot representation of the changes in Chlamydomonas biomass after stress assays. Legend: Control (CTL), Nitrogen (-N), Carbon (-C), and Sulphur (-S) deprivation, Phosphorous limitation (5 % P) deprivations, heat and cold stress (40 °C and 4 °C, respectively), UV radiation stress (UV), salt (0.25 M NaCl) and osmotic (20 % PEG). Gravimetrical measures of pellet weight at the stress phase (48 h) were normalized by its corresponding controls (0 h). Significant changes induced by stress were marked as * (t-test, $p<0.05$) or *** (t-test, $p<0.001$).

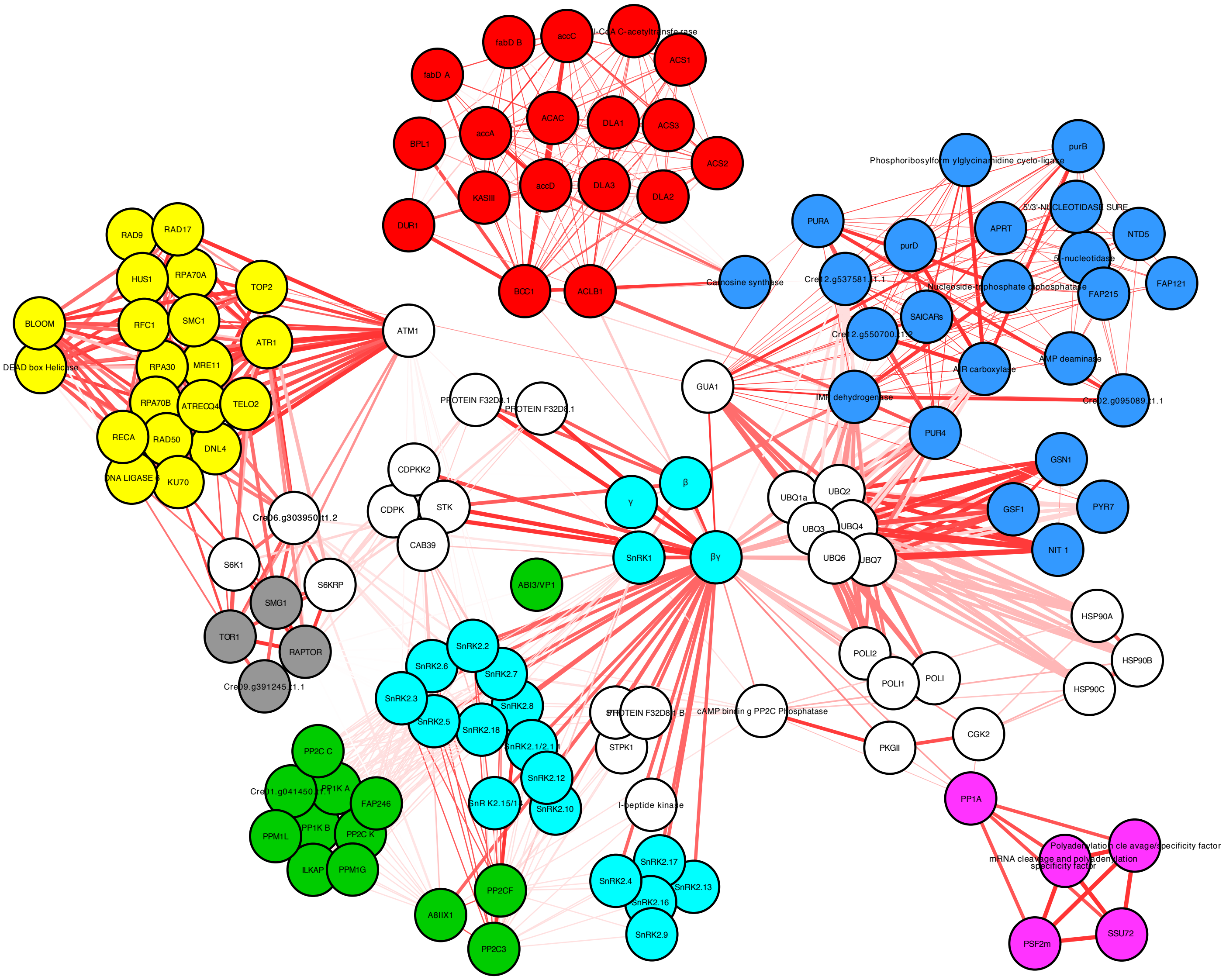

Supplementary Figure S5: STRING based Interaction networks of CKIN proteins. CKINs (cyan) interact with major regulatory groups according to KEGG classification: hormone-signalling response (green), autophagy (grey), DNA repair/transcription (yellow), nitrogen acquisition and derived pathways (dark blue), carbon homeostasis (red), mRNA splicing (purple). White nodes represent bridge proteins, mainly Ca-dependent signalling-related, heat shock, transport, or ubiquitin proteins. Edge thickness represent the combined interaction score provided by STRING-db (confidence range 0.7-0.999) while edge color intensity shows the experimental evidence of the interactions (confidence range 0.131-0.989).

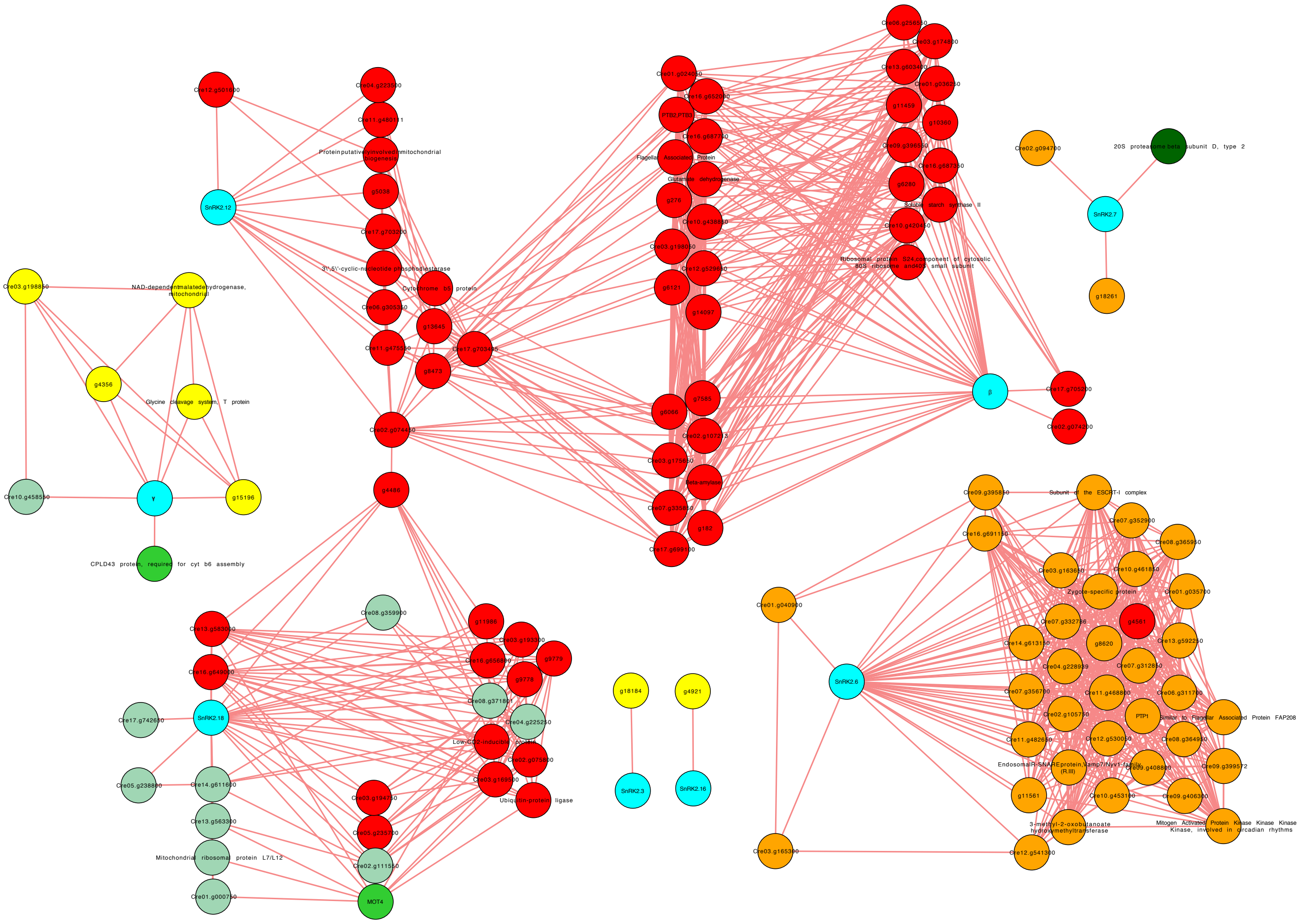

Supplementary Figure S6: ChlamyNet based interaction network of CKIN proteins. CKINs (**cyan**) interact with major regulatory groups according to GO classification: Protein complex assembly and response to misfolded protein (**green**), response to heat and misfolded protein (**dark green**), protein phosphorilation, ribosome biogenesis and macromolecule biosynthesis (**red**), autophagy, GTPase activity and vesicle mediated transport (**orange**), chromatin organization and postranscriptional organization (**yellow**), photosynthesis and hexose metabolic process (**turquoise**).

Supplementary Table S1. *Arabidopsis thaliana* (*Ath*) and *Chlamydomonas reinhardtii* (*Cre*) query sequences used for BLAST homology search to identify putative CKIN sequences into the *C. reinhardtii* genome. Names and accessions (ID) were indicated below. *Chlamydomonas sequences previously referred to as CKIN family by [1] Valledor et al. (2013) and SnRK2 subfamily by [2] Gonzalez-Ballester et al. (2008).

**Name**	***Ath/Cre* ID**	**Name**	***Ath/Cre* ID**	**Name**	***Ath/Cre* ID**
AKIN10	AT3g01090	SnRK3.3	AT4g14580	SnRK3.23	AT1g30270
AKIN11	AT3g29160	SnRK3.4	AT5g57630	SnRK3.24	AT5g10930
β1	AT5g21170	SnRK3.5	AT5g45810	SnRK3.25	AT5g25110
β2	AT4g16360	SnRK3.6	AT5g45820	CKIN1*[1]	Cre04.g211600.t1.1
β3	AT2g28060	SnRK3.7	AT2g34180	CKIN γ*[1]	Cre12.g528000.t1.2
βγ	AT1g09020	SnRK3.8	AT5g58380	CKIN2.1*[2]	Cre02.g075850.t1.1
γ1	AT3g48530	SnRK3.9	AT4g18700	CKIN2.2*[2]	Cre12.g499500.t1.1
γ2	AT1g69800	SnRK3.10	AT3g23000	CKIN2.3*[2]	Cre02.g075900.t1.1
SnRK2.1	AT5g08590	SnRK3.11	AT5g35410	CKIN2.4*[2]	Cre11.g477000.t1.2
SnRK2.2	AT3g50500	SnRK3.12	AT1g01140	CKIN2.5*[2]	Cre03.g209505.t1.1
SnRK2.3	AT5g66880	SnRK3.13	AT4g24400	CKIN2.6*[2]	Cre11.g481000.t1.2
SnRK2.4	AT1g10940	SnRK3.14	AT4g30960	CKIN2.7*[2]	Cre06.g292700.t1.2
SnRK2.5	AT5g63650	SnRK3.15	AT5g01820	CKIN2.8*[2]	Cre10.g466350.t1.1
SnRK2.6	AT4g33950	SnRK3.16	AT3g17510		
SnRK2.7	AT4g40010	SnRK3.17	AT2g26980		
SnRK2.8	AT1g78290	SnRK3.18	AT2g25090		
SnRK2.9	AT2g23030	SnRK3.19	AT2g38490		
SnRK2.10	AT1g60940	SnRK3.20	AT1g29230		
SnRK3.1	AT5g01810	SnRK3.21	AT1g48260		
SnRK3.2	AT5g07070	SnRK3.22	AT2g30360		

Supplementary Table S2. Uncurated BLAST results for *Chlamydomonas* protein. Sequences were considered homologous for e-values lower than 10^{-25}.

Cre Accession	e-value	*Ath* Accession	*Cre* Accession	e-value	*Ath* Accession
Cre10.g457500.t1.1	$1.86e^{-69}$	AT4G16360.1	Cre09.g400330.t1.1	$1.91e^{-37}$	AT1G30270.1
Cre01.g001800.t1.1	$3.71e^{-32}$	AT2G34180.1	Cre09.g400330.t2.1	$1.96e^{-37}$	AT1G30270.1
Cre01.g003524.t1.1	$7.85e^{-37}$	AT4G24400.2	Cre09.g412750.t1.2	$1.91e^{-43}$	AT2G30360.1
Cre01.g009500.t1.2	$1.90e^{-46}$	AT5G35410.1	Cre10.g428650.t1.2	$1.16e^{-45}$	AT5G25110.1
Cre01.g010000.t1.2	$1.31e^{-29}$	AT5G39440.1	Cre10.g443300.t1.2	$5.71e^{-29}$	AT3G01090.1
Cre01.g016570.t1.1	$1.49e^{-29}$	AT2G25090.1	Cre10.g457700.t1.2	$6.47e^{-66}$	AT3G01090.1
Cre01.g029500.t1.2	$1.47e^{-27}$	AT2G26980.3	Cre10.g463500.t1.1	$3.60e^{-27}$	AT5G07070.1
Cre01.g029850.t1.2	$3.03e^{-37}$	AT4G24400.2	Cre10.g464100.t1.2	$6.14e^{-33}$	AT5G25110.1
Cre01.g034900.t1.2	$2.16e^{-30}$	AT3G29160.1	Cre10.g465900.t1.2	$6.17e^{-31}$	AT4G18700.1
Cre01.g036650.t1.1	$3.25e^{-32}$	AT3G01090.1	Cre10.g466350.t1.1	$2.96e^{-152}$	AT4G33950.1
Cre01.g036700.t1.2	$3.30e^{-30}$	AT3G01090.1	Cre11.g467568.t1.1	$8.50e^{-36}$	AT1G29230.1
Cre01.g037100.t1.1	$6.68e^{-34}$	AT1G30270.1	Cre11.g477000.t1.2	$2.35e^{-29}$	AT5G63650.1
Cre01.g048650.t1.1	$9.37e^{-35}$	AT3G01090.1	Cre11.g481000.t1.2	$1.91e^{-88}$	AT4G33950.1
Cre02.g074370.t1.2	$2.51e^{-41}$	AT4G18700.1	Cre12.g484350.t1.1	$2.88e^{-52}$	AT1G09020.1
Cre02.g075350.t1.2	$3.38e^{-33}$	AT3G29160.1	Cre12.g485600.t1.2	$5.64e^{-47}$	AT1G78290.2
Cre02.g075350.t2.1	$2.12e^{-33}$	AT3G29160.1	Cre12.g486350.t1.1	$9.55e^{-38}$	AT3G01090.1
Cre02.g075350.t3.1	$3.38e^{-33}$	AT3G29160.1	Cre12.g493250.t1.2	$8.45e^{-34}$	AT2G30360.1
Cre02.g075850.t1.1	$1.33e^{-57}$	AT4G33950.1	Cre12.g498650.t1.2	$4.16e^{-28}$	AT3G01090.1
Cre02.g075850.t2.1	$1.06e^{-53}$	AT4G33950.1	Cre12.g499500.t1.1	$4.02e^{-117}$	AT1G78290.2
Cre02.g075900.t1.1	$6.60e^{-73}$	AT5G66880.1	Cre12.g499850.t1.1	$1.48e^{-37}$	AT5G25110.1
Cre02.g076000.t1.2	$1.07e^{-40}$	AT5G08590.1	Cre12.g508900.t1.2	$4.55e^{-31}$	AT3G01090.1
Cre02.g076900.t1.1	$5.58e^{-45}$	AT3G23000.1	Cre12.g509000.t1.1	$4.89e^{-32}$	AT3G01090.1
Cre02.g106650.t1.1	$3.39e^{-44}$	AT2G26980.3	Cre12.g514650.t1.2	$6.81e^{-35}$	AT1G30270.1
Cre02.g107800.t1.1	$2.45e^{-32}$	AT3G01090.1	Cre12.g527000.t1.2	$4.34e^{-39}$	AT5G25110.1
Cre02.g112333.t1.1	$1.51e^{-30}$	AT2G30360.1	Cre12.g529550.t1.2	$1.63e^{-36}$	AT1G30270.1
Cre02.g113600.t1.2	$1.12e^{-30}$	AT2G25090.1	Cre12.g537400.t1.1	$1.90e^{-40}$	AT2G30360.1
Cre02.g114750.t1.2	$2.61e^{-48}$	AT1G01140.2	Cre12.g538300.t1.1	$4.36e^{-28}$	AT5G35410.1
Cre03.g144484.t1.1	$5.09e^{-43}$	AT3G01090.1	Cre12.g560350.t1.1	$9.83e^{-34}$	AT5G45820.1
Cre03.g153150.t1.2	$4.07e^{-37}$	AT2G38490.1	Cre12.g560350.t2.1	$9.60e^{-34}$	AT5G45820.1
Cre03.g164900.t1.1	$3.35e^{-27}$	AT2G34180.1	Cre13.g564500.t1.1	$2.17e^{-41}$	AT1G01140.2
Cre03.g169500.t1.2	$4.41e^{-27}$	AT5G45810.1	Cre13.g567550.t1.1	$2.85e^{-32}$	AT1G29230.1
Cre03.g190050.t1.1	$6.79e^{-35}$	AT3G01090.1	Cre13.g567550.t2.1	$3.31e^{-32}$	AT1G29230.1
Cre03.g199050.t1.2	$2.14e^{-49}$	AT1G30270.1	Cre13.g568050.t1.1	$2.59e^{-58}$	AT5G66880.1
Cre03.g209505.t1.1	$3.81e^{-63}$	AT1G78290.2	Cre13.g570250.t1.1	$1.56e^{-76}$	AT3G01090.1
Cre04.g211600.t1.1	0	AT3G01090.1	Cre13.g571700.t1.1	$8.10e^{-52}$	AT3G01090.1
Cre04.g220700.t1.2	$4.92e^{-52}$	AT5G10930.1	Cre13.g579200.t1.2	$2.46e^{-36}$	AT2G25090.1
Cre04.g228800.t1.1	$4.76e^{-29}$	AT1G30270.1	Cre13.g592000.t1.2	$4.10e^{-37}$	AT3G01090.1
Cre05.g232750.t1.2	$9.50e^{-33}$	AT3G01090.1	Cre14.g612000.t1.2	$1.76e^{-35}$	AT1G30270.1
Cre06.g251050.t1.1	$1.44e^{-62}$	AT3G29160.1	Cre16.g657350.t1.2	$5.77e^{-59}$	AT1G78290.2
Cre06.g265550.t1.2	$2.99e^{-43}$	AT5G10930.1	Cre16.g659400.t1.1	$2.12e^{-28}$	AT2G34180.1
Cre06.g268150.t1.1	$2.08e^{-27}$	AT5G39440.1	Cre16.g659400.t2.1	$3.09e^{-29}$	AT1G30270.1
Cre06.g292700.t1.2	$8.68e^{-103}$	AT4G33950.1	Cre16.g663200.t1.1	$1.43e^{-38}$	AT3G29160.1
Cre06.g296200.t1.2	$9.41e^{-46}$	AT3G29160.1	Cre16.g665364.t1.1	$1.16e^{-53}$	AT3G01090.1
Cre06.g306950.t1.1	$1.10e^{-31}$	AT5G01810.1	Cre16.g669800.t1.2	$1.14e^{-50}$	AT5G25110.1
Cre07.g317300.t1.1	$2.83e^{-28}$	AT5G01820.1	Cre16.g672602.t1.1	$2.58e^{-26}$	AT5G35410.1
Cre07.g328850.t1.2	$1.45e^{-28}$	AT1G29230.1	Cre16.g676309.t1.1	$1.32e^{-27}$	AT5G01810.1
Cre07.g328900.t1.2	$3.33e^{-50}$	AT2G26980.3	Cre16.g685389.t1.1	$4.18e^{-37}$	AT4G18700.1
Cre07.g329850.t1.1	$3.09e^{-37}$	AT5G08590.1	Cre17.g705000.t1.2	$7.65e^{-55}$	AT5G25110.1
Cre07.g348450.t1.1	$2.51e^{-30}$	AT5G01810.1	Cre17.g705350.t1.1	$4.72e^{-39}$	AT5G01810.1
Cre07.g351150.t1.2	$1.10e^{-27}$	AT5G45820.1	Cre17.g707800.t1.2	$9.81e^{-46}$	AT1G78290.2
Cre08.g359900.t1.1	$1.71e^{-35}$	AT2G30360.1	Cre17.g719450.t1.1	$4.92e^{-34}$	AT5G10930.1
Cre08.g382800.t1.2	$2.80e^{-35}$	AT5G10930.1	Cre17.g728700.t1.1	$2.71e^{-35}$	AT2G30360.1
Cre08.g385050.t1.1	$2.98e^{-28}$	AT3G01090.1	Cre17.g733300.t1.1	$6.38e^{-33}$	AT4G24400.2
Cre09.g388000.t1.2	$4.15e^{-36}$	AT2G25090.1	Cre17.g747397.t1.1	$5.09e^{-28}$	AT4G30960.1
Cre09.g391245.t1.1	$3.93e^{-47}$	AT1G01140.2	Cre19.g750597.t1.1	$6.08e^{-52}$	AT3G01090.1

Supplementary Table S3. SnRK/CKIN clusters, protein domain names, and domain identifiers (ID) both in *Arabidopsis thaliana* (*Ath*) and *Chlamydomonas reinhardtii* (*Cre*), and corresponding *Cre* sequence names. SnRK clusters according to sequence similarity and protein domain layout (CKIN1/SnRK1/AKIN: containing the Serin/Threonin Kinase PTHR24343:SF183, IPR015940 and IPR001772 domains; S1R: regulatory subunits of SnRK1 with PTHR10343, PTHR13780:SF35, IPR032640, IPR000644, IPR006828 and IPR013785 domains; CKIN2/SnRK2: containing the Serin/Threonin Kinase PTHR24343, PTHR24343:SF169, PTHR24343:SF207, PTHR24343:SF167 or PTHR24343:SF200 domains, with CKIN1L exception, containing the MAP/microtubule affinity-regulating kinase PTHR24346:SF5 or the Serin/Threonin protein kinase PTHR24343:SF302 and IPR015940 domains).

SnRK Cluster	Domain name	*Ath* Domain ID	*Cre* Domain ID	*Cre* Name
CKIN1/SnRK1/AKIN	KA1 domain	IPR001772	IPR001772	CKIN1
	Serin/Threonin Kinase	PTHR24343	PTHR24343	CKIN1
	Serin/Threonin Kinase	PTHR24343:SF183	PTHR24343:SF183	CKIN1
	Serin/Threonin Kinase	PTHR24343:SF156		
	UBA domain	IPR015940	IPR015940	CKIN1
	MAP/microtubule affinity-regulating		PTHR24346	
	kinase		PTHR24346:SF5	CKIN1L
	Serin/Threonin protein kinase		PTHR24343:SF302	
	UBA domain			CKIN1L
			IPR015940	CKIN1L
S1 R (β)	AMPK, β subunit	PTHR10343	PTHR10343	CKIN β
	SnRK 1, β-1 regulatory subunit	PTHR10343:SF51		CKIN β
	Immunoglobulin E-set	IPR014756	IPR014756	CKIN β
	AMPK, glycogen-binding domain	IPR032640	IPR032640	CKIN β
	Association with the SNF1 complex domain (ASC)	IPR006828	IPR006828	CKIN β
S1 R (βγ)	AMPK, γ regulatory subunit	PTHR13780	PTHR13780	CKIN βγ
	AMPK, γ regulatory subunit		PTHR13780:SF35	CKIN βγ
	AMP-activated protein kinase, glycogen-binding domain	IPR032640	IPR032640	CKIN βγ
	CBS domain	IPR000644	IPR000644	CKIN βγ
	Immunoglobulin E-set	IPR014756	IPR014756	CKIN βγ
S1 R (γ)	AMPK, γ regulatory subunit	PTHR13780	PTHR13780	CKIN γ
	AMPK, γ regulatory subunit	PTHR13780:SF47		
	SnRK 1, γ regulatory subunit	PTHR13780:SF36		
	AMPK, γ regulatory subunit		PTHR13780:SF49	CKIN γ
	CBS domain	IPR000644	IPR000644	CKIN γ
CKIN2/SnRK2	Serin/Threonin kinase	PTHR24343	PTHR24343	CKIN2
	Serin/Threonin Protein Kinase SRK2D-Related		PTHR24343:SF169	CKIN2.8
	Serin/Threonin Protein Kinase SRK2C		PTHR24343:SF207	CKIN2.2
	Serin/Threonin Protein Kinase SRK2E		PTHR24343:SF167	CKIN2.12
				CKIN2.19
				CKIN2.10
				CKIN2.13
CKINL	Serin/Threonin Protein Kinase SRK2F		PTHR24343:SF200	CKINL
SnRK3	Serin/Threonin Kinase	PTHR24343	PTHR24343	
	Calcium/calmodulin-dependent/calcium-dependent protein kinase	PTHR24347	PTHR24347	
	NAF	IPR004041		
	NAF/FISL	IPR018451		

Supplementary Table S4. *Arabidopsis thaliana* (*Ath*), *Chlamydomonas reinhardtii* (*Cre*), *Chlorella variabilis* (*Cva*), *Coccomyxa subellipsoidea* (*Csu*), *Dunaliella salina* (*Dsa*) and *Volvox carteri* (*Vca*) sequences used for the design of the figure 3 sequence tree: sequence identifier (Seq ID) followed by sequence name (Name).

Seq ID	Name	Seq ID	Name
AT3g01090	*Ath* AKIN10	jgi|ChlNC64A_1|140361|IGS.gm_3_00100	*Cva* CvKIN2.10
AT3g29160	*Ath* AKIN11	jgi|ChlNC64A_1|142950|IGS.gm_5_00279	*Cva* CvKIN2.11
AT5g39440	*Ath* SnRK1.3	jgi|ChlNC64A_1|143933|IGS.gm_6_00507	*Cva* CvKIN2.12
AT5g08590	*Ath* SnRK2.1	jgi|ChlNC64A_1|145069|IGS.gm_8_00397	*Cva* CvKIN2.13
AT3g50500	*Ath* SnRK2.2	jgi|ChlNC64A_1|140646|IGS.gm_3_00385	*Cva* CvKIN2.14
AT5g66880	*Ath* SnRK2.3	e_gw1.10.111.1|16775	*Csu* CsKIN1
AT4g33950	*Ath* SnRK2.6	e_gw1.3.241.1|12786	*Csu* CsKIN2.2
AT4g40010	*Ath* SnRK2.7	estExt_fgenesh1_pg.C_100215|47867	*Csu* CsKIN2.3
AT2g23030	*Ath* SnRK2.9	estExt_fgenesh1_pg.C_240007|49286	*Csu* CsKIN2.4
AT1g60940	*Ath* SnRK2.10	estExt_fgenesh1_pm.C_150088|54361	*Csu* CsKIN2.5
AT5g01810	*Ath* SnRK3.1	estExt_Genemark1.C_30088|64800	*Csu* CsKIN2.6
AT5g07070	*Ath* SnRK3.2	estExt_Genemark1.C_30287|64904	*Csu* CsKIN2.7
AT4g14580	*Ath* SnRK3.3	estExt_Genemark1.C_90162|66347	*Csu* CsKIN2.8
AT5g57630	*Ath* SnRK3.4	estExt_Genemark1.C_240049|68309	*Csu* CsKIN2.9
AT5g45810	*Ath* SnRK3.5	estExt_Genewise1.C_100280|24306	*Csu* CsKIN2.10
Cre04.g211600.t1.1	*Cre* CKIN1	estExt_Genewise1.C_190274|25912	*Csu* CsKIN2.11
Cre13.g570250.t1.1	*Cre* CKIN1L	estExt_Genewise1Plus.C_30441|27599	*Csu* CsKIN2.12
Cre02.g075850.t1.1	*Cre* CKIN2.1	estExt_Genewise1Plus.C_30509|27636	*Csu* CsKIN2.13
Cre12.g499500.t1.1	*Cre* CKIN2.2	estExt_Genewise1Plus.C_30548|27662	*Csu* CsKIN2.14
Cre02.g075900.t1.1	*Cre* CKIN2.3	estExt_Genewise1Plus.C_100351|29722	*Csu* CsKIN2.15
Cre11.g477000.t1.2	*Cre* CKIN2.4	estExt_Genewise1Plus.C_100354|29725	*Csu* CsKIN2.16
Cre03.g209505.t1.1	*Cre* CKIN2.5	fgenesh1_pg.20_#_52|44853	*Csu* CsKIN2.17
Cre11.g481000.t1.2	*Cre* CKIN2.6	e_gw1.5.212.1|14400	*Csu* CsKIN2.18
Cre06.g292700.t1.2	*Cre* CKIN2.7	Dusal.0281s00020|Dusal.0281s00020.1	*Dsa* DKIN1
Cre10.g466350.t1.1	*Cre* CKIN2.8	Dusal.0029s00005|Dusal.0029s00005.1	*Dsa* DKIN2.2
Cre13.g568050.t1.3	*Cre* CKIN2.14	Dusal.0056s00014|Dusal.0056s00014.1	*Dsa* DKIN2.3
Cre16.g657350.t1.2	*Cre* CKIN2.9	Dusal.0106s00018|Dusal.0106s00018.1	*Dsa* DKIN2.5
Cre17.g707800.t1.2	*Cre* CKIN2.10	Dusal.0181s00032|Dusal.0181s00032.1	*Dsa* DKIN2.6
Cre12.g485600.t1.2	*Cre* CKIN2.13	Dusal.0186s00019|Dusal.0186s00019.1	*Dsa* DKIN2.7
Cre02.g076000.t1.2	*Cre* CKIN2.11	Dusal.0617s00007|Dusal.0617s00007.1	*Dsa* DKIN2.8
Cre08.g384250.t1.2	*Cre* CKIN2.15	Dusal.0826s00006|Dusal.0826s00006.1	*Dsa* DKIN2.9
Cre17.g707650.t1.1	*Cre* CKIN2.12	fgenesh1_pm.C_Chr_6000070|12650	*Olu* OKIN1
Cre07.g329850.t1.1	*Cre* CKIN2.16	eugene.0100010317|28923	*Olu* OKIN2.2
Cre02.g074370.t1.2	*Cre* CDPK1	ost_03_006_039|86646	*Olu* OKIN2.3
Cre02.g106650.t1.3	*Cre* CDPK2	Vocar.0025s0127.1	*Vca* VKIN1
Cre06.g265550.t1.2	*Cre* CDPK3	Vocar.0048s0006|Vocar.0048s0006.1	*Vca* VKIN2.2
Cre06.g296200.t1.2	*Cre* CDPK4	Vocar.0027s0049|Vocar.0027s0049.1	*Vca* VKIN2.3
Cre07.g328900.t1.2	*Cre* CDPK5	Vocar.0031s0053|Vocar.0031s0053.1	*Vca* VKIN2.5
Cre01.g048650.t1.1	*Cre* CDPK6	Vocar.0004s0065|Vocar.0004s0065.1	*Vca* VKIN2.6
Cre13.g564500.t1.3	*Cre* CDPK8	Vocar.0021s0079|Vocar.0021s0079.1	*Vca* VKIN2.7
Cre13.g571700.t1.1	*Cre* CDPK9	Vocar.0029s0126|Vocar.0029s0126.1	*Vca* VKIN2.8
Cre19.g750597.t1.1	*Cre* CDPK11	Vocar.0013s0156|Vocar.0013s0156.1	*Vca* VKIN2.9
Cre01.g003524.t1.1	*Cre* CDPK12	Vocar.0024s0254|Vocar.0024s0254.1	*Vca* VKIN2.10
jgi|ChlNC64A_1|57611|estExt_fgenesh3_pg.C_80094	*Cva* CvKIN2.1	Vocar.0006s0284|Vocar.0006s0284.1	*Vca* VKIN2.11
jgi|ChlNC64A_1|33664|estExt_Genewise1Plus.C_10631	*Cva* CvKIN2.3	Vocar.0001s0337.1	*Vca* VKIN2.12
jgi|ChlNC64A_1|34706|estExt_Genewise1Plus.C_50310	*Cva* CvKIN2.4	Vocar.0001s1516|Vocar.0001s1516.1	*Vca* VKIN2.13
jgi|ChlNC64A_1|57249|estExt_fgenesh3_pg.C_50159	*Cva* CvKIN2.5	Vocar.0001s1602|Vocar.0001s1602.1	*Vca* VKIN2.14
jgi|ChlNC64A_1|135543|IGS.gm_14_00076	*Cva* CvKIN2.6	Vocar.0001s1603|Vocar.0001s1603.1	*Vca* VKIN2.15
jgi|ChlNC64A_1|138144|IGS.gm_2_00611	*Cva* CvKIN2.8	Vocar.0001s1604|Vocar.0001s1604.1	*Vca* VKIN2.16
jgi|ChlNC64A_1|138887|IGS.gm_22_00128	*Cva* CvKIN2.9		

Supplementary Table S5: Fold-change variations in the abundance of the different members of the CKIN family on data taken from Algaepath (a), and under the different stress situations that were tested by qPCR (control vs 48 h of stress).

a) Algaepath

Name	S-	N-	CO_2--	H_2O_2, 1.0h	Fe--
CKIN1	1.77	0.89	2.69	0.57	1.32
CKIN1L	2.18	0.79	2.10	0.64	9.49
CKIN β	1.30	0.54	1.78	0.59	0.72
CKIN βγ	2.33	1.40	2.03	0.51	1.17
CKIN γ	0.93	0.40	0.60	0.96	0.58
CKIN2.1	1.23	0.96	1.39	0.91	1.05
CKIN2.2	4.11	2.41	1.24	1.58	1.31
CKIN2.3	2.60	1.39	1.94	0.76	1.15
CKIN2.4	1.44	0.60	1.04	0.82	0.81
CKIN2.5	3.52	1.38	1.23	1.06	1.49
CKIN2.6	2.56	1.47	1.29	1.26	2.08
CKIN2.7	1.59	0.87	1.69	2.55	1.20
CKIN2.8	1.60	1.11	1.39	1.51	1.68
CKIN2.9	2.13	1.28	1.01	0.98	0.77
CKIN2.10	1.77	1.24	1.62	1.02	1.01
CKIN2.11	2.00	1.18	1.84	0.63	1.66
CKIN2.12	2.32	0.77	1.26	1.25	1.75
CKIN2.13	1.60	1.58	3.45	0.77	7.82
CKIN2.14	0.75	0.33	1.42	1.27	2.28
CKIN2.15	4.48	2.40	1.01	2.87	8.00
CKIN2.16	3.03	1.40	1.86	1.52	1.82
CKINL	16.04	5.61	1.82	0.48	0.69

b) qPCR assays

Name	S-	N-	C-	5 % P	20 % PEG	0.25M NaCl	40 °C	4 °C	UV
CKIN1	2.08	0.75	1.72	11.98	3.38	18.61	3.28	4.54	2.08
CKIN1L	2.94	0.41	1.35	1.04	0.64	1.87	2.59	1.03	1.52
CKIN β	1.42	0.33	1.37	8.03	8.33	8.70	2.15	3.21	7.53
CKIN βγ	5.23	1.72	1.12	4.05	3.35	10.18	3.57	8.97	6.32
CKIN γ	1.37	0.51	2.11	9.31	1.28	16.03	3.75	6.77	5.40
CKIN2.1	1.00	0.76	0.67	1.33	440.00	6.72	2.75	13.54	21.95
CKIN2.2	11.62	2.72	1.09	1.98	5.15	6.59	2.83	13.64	9.07
CKIN2.3	4.73	1.34	1.51	3.03	8.74	12.74	2.77	9.95	5.79
CKIN2.4	1.64	0.42	1.22	0.94	29.80	2.21	9.16	2.91	1.91
CKIN2.5	10.38	1.50	1.17	5.16	105.61	14.33	6.72	8.30	21.22
CKIN2.6	4.44	1.12	0.95	0.80	8.97	1.60	1.22	1.86	13.63
CKIN2.7	2.15	0.65	1.05	2.09	8.05	9.10	2.69	6.26	25.96
CKIN2.8	1.74	0.62	0.69	0.19	21.20	4.09	2.83	2.47	10.69
CKIN2.9	4.99	1.13	0.46	1.27	16.89	2.78	1.74	2.12	9.12
CKIN2.10	1.98	1.16	2.61	2.60	71.84	7.35	4.82	2.20	22.04
CKIN2.11	2.45	1.17	0.49	0.72	134.08	7.97	1.78	5.87	19.37
CKIN2.12	4.47	1.16	0.58	1.38	354.10	5.94	1.25	6.46	7.76
CKIN2.13	1.87	0.44	0.67	0.37	6.71	9.19	1.47	9.78	3.37
CKIN2.14	0.28	0.10	0.25	0.16	1.64	0.48	2.16	0.38	0.52
CKIN2.15	13.77	2.21	0.91	1.39	230.70	5.73	2.65	6.67	10.71
CKIN2.16	6.87	1.88	1.02	1.20	3.52	4.24	2.22	1.78	6.99
CKINL	82.94	16.79	0.27	0.94	31.78	10.65	7.31	10.78	5.90

Supplementary Table S6. Sequences of *Arabidopsis thaliana* (*Ath*), *Saccharomyces cerevisiae* (*Sce*), *Homo sapiens* (*Hsa*), and *Chlamydomonas reinhardtii* (*Cre*) employed for building sequence trees. Organism identification followed by sequence name (Name) and sequence identifier (Seq ID).

Name	Seq ID	Name	Seq ID
Ath AKIN10	AT3g01090	*Cre* CKIN2.6	Cre11.g481000.t1.2
Ath AKIN11	AT3g29160	*Cre* CKIN2.7	Cre06.g292700.t1.2
Ath SnRK1.3	AT5g39440	*Cre* CKIN2.8	Cre10.g466350.t1.1
Hsa AMPK α1	NP_006242.5	*Cre* CKIN2.9	Cre16.g657350.t1.2
Cre CKIN1	Cre04.g211600.t1.1	*Cre* CKIN2.10	Cre17.g707800.t1.2
Ath β1	AT5g21170	*Cre* CKIN2.11	Cre02.g076000.t1.2
Hsa AMPK β2	NP_005390.1	*Cre* CKIN2.12	Cre17.g707650.t1.1
Cre CKIN1L	Cre13.g570250.t1.1	*Cre* CKIN2.13	Cre12.g485600.t1.2
Sce SNF1	NP_010765.3	*Cre* CKIN2.14	Cre13.g568050.t1.3
Ath β2	AT4g16360	*Cre* CKIN2.15	Cre08.g384250.t1.2
Cre CKIN β	Cre10.g457500.t1.1	*Cre* CKIN2.16	Cre07.g329850.t1.1
Ath βγ	AT1g09020	*Cre* CKINL	Cre16.g685389.t1.1
Hsa AMPK γ1	NP_002724.1	*Ath* SnRK3.1	AT5g01810
Cre CKIN βγ	Cre12.g484350.t1.3	*Ath* SnRK3.2	AT5G07070
Ath γ1	AT3g48530	*Ath* SnRK3.3	AT4g14580
Ath γ2	AT1g69800	*Ath* SnRK3.4	AT5g57630
Cre CKIN γ	Cre12.g528000.t1.2	*Ath* SnRK3.5	AT5g45810
Ath SnRK2.2	AT3g50500	*Cre* CDPK1	Cre02.g074370.t1.2
Ath SnRK2.6	AT4g33950	*Cre* CDPK2	Cre02.g106650.t1.3
Ath SnRK2.7	AT4g40010	*Cre* CDPK3	Cre06.g265550.t1.2
Ath SnRK2.9	AT2g23030	*Cre* CDPK4	Cre06.g296200.t1.2
Ath SnRK2.10	AT1g60940	*Cre* CDPK5	Cre07.g328900.t1.2
Cre CKIN2.1.	Cre02.g075850.t1.1	*Cre* CDPK6	Cre01.g048650.t1.1
Cre CKIN2.2	Cre12.g499500.t1.1	*Cre* CDPK8	Cre13.g564500.t1.3
Cre CKIN2.3	Cre02.g075900.t1.1	*Cre* CDPK9	Cre13.g571700.t1.1
Cre CKIN2.4	Cre11.g477000.t1.2	*Cre* CDPK11	Cre19.g750597.t1.1
Cre CKIN2.5	Cre03.g209505.t1.1	*Cre* CDPK12	Cre01.g003524.t1.1

Supplementary Table S7. Primer pairs used for RT-qPCR analyses. All primers were designed with Tm greater than 60°C and PCR were all conducted at an annealing temperature of 61°C.

Name	ID	Forward primer sequence	Reverse primer sequence
ACT	Cre13.g603700.t1.2	GCGGCTACTCGTTCACCACCAC	TCTCCTGCTCGAAGTCCAGGGC
UBI	Cre03.g159200.t1.1	CTACCGCCGTTCCTGTTCCTGC	CTGGCGGCAGTAGCACCACATC
TUB	Cre04.g216850.t1.2	GTCCAAGCTGGGCTTCACCGTC	GGCGGCAGATGTCGTAGATGGC
RCK1	Cre06.g278222.t1.1	CGACAAGAGCGTGCTGGTCTGG	GTCAGGCAGAACTGGCCATCGG
CKIN1	Cre04.g211600.t1.1	TCATGCACCCGCACATCATCCG	CAGCCGCCCCTTCTCCACAATG
CKIN1L	Cre13.g570250.t1.1	CGTGGCCAGCCTCGAGTACTGC	GGGCACGTAGAAGGCGGACAGG
CKIN β	Cre10.g457500.t1.1	GATGAGCTGACAGCCGCCAACC	CACCGTGGCTCCACACGATGAC
CKIN βγ	Cre12.g484350.t1.3	ACGGCTCTCCTGGGTTGTTTGC	TTGACGTTGCCAAGCGGGTCTG
CKIN γ	Cre12.g528000.t1.2	CGTGCTGATGCAGGAGCTGGAG	TCACGGTGTGCACCTTCTTGGC
CKIN2.1	Cre02.g075850.t1.1	GGTCAAGCGTGAAGTGCGAACC	GCAGTCCGCGTACTCCATCACC
CKIN2.2	Cre12.g499500.t1.1	CATCTGGAGCTGCGGCGTGATG	GCGGCGGGATGTGGTAGTCAAC
CKIN2.3	Cre02.g075900.t1.1	CGTGTTCGGCCTCGACTACTGC	GCAGGTTGAAGGGGTGCTCCAG
CKIN2.4	Cre11.g477000.t1.2	GCGCGAAATTCAGTCCCATCGC	CCCTGGTCAGCGAACTCCATGC
CKIN2.5	Cre03.g209505.t1.1	CGGTGGACTACTGCCACAAGCG	GGACCTGAAGTCGGCCTTGCTG
CKIN2.6	Cre11.g481000.t1.2	GGCGGGAGCTTGTTCCACTACG	TCCAGCTTGATGTCGCGGTTGG
CKIN2.7	Cre06.g292700.t1.2	GCTGCCGCTGCTCAAAATCTGC	GTACTGGTCCGTGGCGCGAATC
CKIN2.8	Cre10.g466350.t1.1	GTGTGCCACCGGGATCTGAAGC	TCTTGGGCTGGCTGTCAAACGC
CKIN2.9	Cre16.g657350.t1.2	GGACGAGGCGCGGTACTTCTTC	GTCGCACAGCTTCAGCCAGGAG
CKIN2.10	Cre17.g707800.t1.2	GGCCTGCAGCGAGGAGTTCAA	GCGGTTGAAGGTGGGGTAGTGC
CKIN2.11	Cre02.g076000.t1.2	ACCTGAAGCGCGAGGTGGTGTG	GCTCCGCCAGCCACTGCTTGAG
CKIN2.12	Cre17.g707650.t1.1	GGTGATGGAGCTGCTGGGCACAG	GCTTGCAGCTGCAGCTCCATGC
CKIN2.13	Cre12.g485600.t1.2	AGGACGAGCACAAGCGCATCA	ACGGGTGGCCAGGTCCTGTATG
CKIN2.14	Cre13.g568050.t1.3	CTGGAGCCGCGGGTTCTACAAG	TCCATCACCACCGCCAGGTAGG
CKIN2.15	Cre08.g384250.t1.2	CATCTGGTCCTGCGGCGTGGTG	TCCTCGCGCGGCACCATGATG
CKIN2.16	Cre07.g329850.t1.1	CATGTCGGCCGCCTCCCTTGAC	CGCGTGCGGCAGATGGTGTAGG
CKINL	Cre16.g685389.t1.1	GACGTCGCCCATGGGTCAGCAC	GGAGGTGCTACTGCCGGCATCC
CHX	Cre04.g215050.t1.2	ATGATGCTGGCTTCGCGTCCTG	CACAATGGGGTCCGCGACCTTC
